# Noninferiority of Silver Diamine Fluoride vs Sealants for Reducing Dental Caries Prevalence and Incidence: A Randomized Clinical Trial

**DOI:** 10.1001/jamapediatrics.2023.6770

**Published:** 2024-03-04

**Authors:** Ryan Richard Ruff, Tamarinda J. Barry Godín, Richard Niederman

**Affiliations:** 1 Department of Epidemiology & Health Promotion, New York University College of Dentistry, New York; 2 New York University School of Global Public Health, New York

## Abstract

**Importance:**

Dental caries is the world’s most prevalent noncommunicable disease and a source of health inequity; school dental sealant programs are a common preventive measure. Silver diamine fluoride (SDF) may provide an alternative therapy to prevent and control caries if shown to be noninferior to sealant treatment.

**Objective:**

To determine whether school-based application of SDF is noninferior to dental sealants and atraumatic restorative treatment (ART) in the prevalence of dental caries.

**Design, Setting, and Participants:**

The Silver Diamine Fluoride Versus Therapeutic Sealants for the Arrest and Prevention of Dental Caries in Low-Income Minority Children (CariedAway) study was a pragmatic noninferiority cluster-randomized clinical trial conducted from February 2018 to June 2023 to compare silver diamine fluoride vs therapeutic sealants for the arrest and prevention of dental caries. Children at primary schools in New York, New York, with at least 50% of the student population reporting as Black or Hispanic and at least 80% receiving free or reduced lunch were included. This population was selected as they are at the highest risk of caries in New York. Students were randomized to receive either SDF or sealant with ART; those aged 5 to 13 years were included in the analysis. Treatment was provided at every visit based on need, and the number of visits varied by child. Schools with preexisting oral health programs were excluded, as were children who did not speak English. Of 17 741 students assessed for eligibility, 7418 were randomized, and 4100 completed follow-up and were included in the final analysis.

**Interventions:**

Participants were randomized at the school level to receive either a 38% concentration SDF solution or glass ionomer sealants and ART. Each participant also received fluoride varnish.

**Main Outcomes and Measures:**

Primary study outcomes were the prevalence and incidence of dental caries.

**Results:**

A total of 7418 children (mean [SD] age, 7.58 [1.90] years; 4006 [54.0%] female; 125 [1.7%] Asian, 1246 [16.8%] Black, 3648 [49.2%] Hispanic, 153 [2.1%] White, 114 [1.5%] multiple races or ethnicities, 90 [1.2%] other [unspecified], 2042 [27.5%] unreported) were enrolled and randomized to receive either SDF (n = 3739) or sealants with ART (n = 3679). After initial treatment, 4100 participants (55.0%) completed at least 1 follow-up observation. The overall baseline prevalence of dental caries was approximately 27.2% (95% CI, 25.7-28.6). The odds of decay prevalence decreased longitudinally (odds ratio [OR], 0.79; 95% CI, 0.75-0.83) and SDF was noninferior compared to sealants and ART (OR, 0.94; 95% CI, 0.80-1.11). The crude incidence of dental caries in children treated with SDF was 10.2 per 1000 tooth-years vs 9.8 per 1000 tooth-years in children treated with sealants and ART (rate ratio, 1.05; 95% CI, 0.97-1.12).

**Conclusions and Relevance:**

In this school-based pragmatic randomized clinical trial, application of SDF resulted in nearly identical caries incidence compared to dental sealants and ART and was noninferior in the longitudinal prevalence of caries. These findings suggest that SDF may provide an effective alternative for use in school caries prevention.

**Trial Registration:**

ClinicalTrials.gov Identifier: NCT03442309

## Key Points

**Question:**

Is silver diamine fluoride noninferior to dental sealants and atraumatic restorations in the prevalence of dental caries when used in schools?

**Findings:**

In this randomized clinical trial of 7418 children, the incidence of dental caries was comparable between children treated with silver diamine fluoride and those treated with dental sealants and atraumatic restorations. For caries prevalence, silver diamine fluoride was noninferior in adjusted models.

**Meaning:**

The findings suggest that silver diamine fluoride can be effectively used as a primary intervention for school-based caries prevention.

## Teaser

This randomized clinical trial evaluates the use of silver diamine fluoride for reducing the prevalence of dental caries.

## Introduction

Dental caries is the world’s most prevalent noncommunicable disease.^1^ The National Institute of Dental and Craniofacial Research^2^ estimates that more than 50% of US children between the ages of 6 and 8 years have experienced caries, with the rate in some racial and ethnic minority groups exceeding 70%. The United Nations considers oral diseases to be a major global burden that shares common risk factors with other noncommunicable diseases, and the World Health Organization Global Oral Health Action Plan^3^ names oral disease prevention as a primary strategic objective, recommending the use of cost-effective, community-based methods to prevent caries. In 2022, the World Health Organization added glass ionomer sealants and silver diamine fluoride (SDF) to its Model List of Essential Medicines.^4^

Despite increases in Medicaid entitlements for dental benefits, there remain persistent access challenges to oral disease prevention throughout the US. More than 69 million individuals in the US live in dental care health professional shortage areas.^5^ The US Centers for Disease Control and Prevention^6^ recommends school sealant programs to increase access to care and improve health equity. Dental sealants—thin coatings applied to the pits and fissures of teeth to protect against bacteria—can prevent the onset of carious lesions and arrests them in the early stages.^7,8^ However, the burgeoning costs of care limits their use in schools.^9^ Alternatively, SDF is a colorless alkaline solution consisting of silver and fluoride with antimicrobial properties that remineralizes teeth. In clinical studies, SDF application prevents caries in the primary dentition compared to placebo^10^ and arrests existing caries.^11^ SDF can be applied in minutes^12^ and is a cost-effective strategy to reduce the burden of caries, particularly in under resourced areas.^13^ In 2017, the US Food and Drug Administration^14^ granted breakthrough therapy status to SDF.

The Silver Diamine Fluoride Versus Therapeutic Sealants for the Arrest and Prevention of Dental Caries in Low-Income Minority Children (CariedAway) pragmatic^15^ randomized clinical trial^16^ investigated the use of SDF as an alternative therapy for community-based caries control and prevention. Primary clinical outcomes for CariedAway included the noninferiority of SDF compared to dental sealants and atraumatic restorative treatment (ART) in the 2-year arrest of existing dental caries^17^ and the 4-year prevalence of caries. We report on the cumulative incidence and prevalence of caries over 4 years.

## Methods

### Design and Participants

CariedAway was a longitudinal noninferiority pragmatic cluster-randomized clinical trial^16^ conducted from February 1, 2019, to June 1, 2023. The trial was approved by the New York University School of Medicine Institutional Review Board, is registered at ClinicalTrials.gov (NCT03442309), and is reported following the Enhancing the Quality and Transparency of Health Research (EQUATOR) reporting guideline. The trial protocol is Supplement 1.

Any school in the New York, New York, metropolitan area with a total student population consisting of at least 50% Black or Hispanic students and with at least 80% of students receiving free or reduced lunch was eligible for inclusion. This population was selected as they are at the highest risk of caries in New York City. For enrolled schools, any child with parental written informed consent and participant assent was enrolled. While any child meeting these criteria was enrolled, inclusion into analysis was restricted to those aged 5 to 13 years. Additional exclusion criteria were if the school had a preexisting oral health program or if the child did not speak English.

### Interventions and Procedures

Our primary experimental condition consisted of a 38% SDF solution (2.24 F-ion mg/dose). The active control consisted of glass ionomer cement sealants and atraumatic restorations, a minimally invasive approach that consists of preventive and restorative components to halt the progression of caries.^18,19^ Each participant also received a 5% sodium fluoride application.

For the experimental treatment, petroleum jelly was first applied to the lips and surrounding skin to prevent temporary staining that can result from direct contact of SDF with the soft tissue. Isolation was achieved by placing gauze and cotton rolls between the teeth to be treated and the tongue and cheek. One to 2 drops of SDF were dispensed into a mixing well and applied using a microapplicator to all posterior asymptomatic cavitated lesions as well as pits and fissures of premolars and molars. The material was agitated on the surface of all cavities using a scrubbing motion for a minimum of 30 seconds, followed by 60 seconds of air-drying time. One unit dose of fluoride varnish was then applied to all teeth to mask the taste of the SDF. The procedure was then repeated every 6 months per protocol with the exception of the disruption in study activities due to COVID-19.

For the active control, cavity conditioner was first applied to pits and fissures for 10 seconds. Sealant capsules were mixed for 10 seconds at 4000 rpm and then applied directly via the finger-sweep technique to all pits and fissures of bicuspids and molars, ensuring that closed margins were achieved. Atraumatic restorations were also placed on asymptomatic cavitated lesions, and fluoride varnish was applied to all teeth. At successive observations, sealants were reapplied to any unsealed or partially sealed bicuspids and molars. All treatments in both groups were provided in a dedicated room in each school using mobile equipment. Treatments in both groups were continued as long as the child was enrolled in the study.

### Examiners

Treatments in the experimental group were provided by either dental hygienists or registered nurses, and by dental hygienists in the active control. All dental hygienists and registered nurses received identical training prior to the start of the academic year. Training consisted of didactic and experiential activities including screening, treatment protocol standardization exercises, and mock patient interactions.

### Data Collection and Outcomes

Primary outcomes were the prevalence and incidence of dental caries. Caries diagnosis was determined through a full visual-tactile oral examination following the International Caries Detection and Assessment System (ICDAS) adapted criteria for epidemiology and clinical research settings.^20^ Each tooth surface was assessed as being either intact/sound, sealed, restored, decayed, or arrested. Screening criteria considered lesions scored as a 5 (distinct cavity with visible dentin) or 6 (extensive, more than half the surface, distinct cavity with visible dentin) on the ICDAS scale as decay. Any clinical presentation of a filling (eg, amalgam, composite, or stainless steel crown) on a tooth that previously was recorded as sound was analyzed as decay, as it indicated disease incidence occurring between observations.

Demographic data, including sex, race, and ethnicity, were obtained prior to treatment for future analyses. Race and ethnicity data were collected via self-report to support future assessment of effects within sociodemographic groups. Categories were derived from the New York City Department of Education. Participants selecting “other” race or ethnicity included all those not included in specific categories.

### Randomization and Blinding

Schools were block-randomized to either the experimental or active control arm using a random number generator performed by one of us (R. R. R.) and verified by another (T. B. G.). Due to the staining effect of SDF, patients would be able to derive their groups. Clinicians were not blinded as the procedures differed for each treatment; however, they were not able to discern who treated each participant at prior study observations.

### Impact of COVID-19

Data in CariedAway were collected biannually. However, due to the impact of COVID-19, schools in New York City were closed to health care professionals from March 2020 through September 2021. The time elapsed between observations corresponding to this period was approximately 2 years.

### Power

Sample size calculations for the longitudinal prevalence of caries were previously reported^16^ assuming a power of 0.80, error rate of 5%, a repeated measures correlation of 0.5, and a per-visit attrition rate of 20%. Estimates also assumed a minimally detectable effect size of 0.25 and an intraclass correlation coefficient of 0.10, yielding a sample size of 12 874. However the observed intraclass correlation coefficient, computed using intercept-only mixed effects models, ranged from 0.013 (prevalence) to 0.015 (incidence). Thus, the final participant enrollment was sufficient for power requirements.

### Statistical Analysis

At each observation, the proportion of participants in treatment groups with new caries or fillings was determined and bootstrapped confidence intervals for the difference were computed, accounting for any clustering effect of schools. We assessed longitudinal noninferiority using mixed-effects logistic regression models, where the outcome was the presence or absence of any new caries at each observation. Models included random intercepts for individual participants and school. Our noninferiority margin (0.10) was previously selected based on historical evidence and clinical judgement as to what would be an acceptable difference in efficacy for the prevention of dental caries.^21,22^ We converted the margin to the odds ratio (OR) scale by taking the average of the success proportion in the active control arm and determining the equivalent margin (OR δ, 0.63).^23^ We tested noninferiority at any observation by including an interaction between treatment and time, followed by a model with no interaction to assess noninferiority marginally. Comparisons to the OR δ were made using a (1 − 2α) confidence interval for the effect of treatment.^24^

We calculated the incidence rate for the total number of individual teeth that developed caries (in tooth-years) and derived the rate ratio as the most efficient estimator due to the small degree of intracluster correlation in responses.^25^ We then modeled the per-person number of caries present at each observation using multilevel mixed-effects negative binomial regression. Prespecified subgroup analyses for the effect of treatment over time and by the presence or absence of caries at baseline were performed.

We also conducted a series of supplementary analyses. To account for censored observations, we analyzed caries incidence using Cox proportional hazards regression with nonparametric maximum likelihood estimation.^26^ We then assessed whether treatment by either a dental hygienist or registered nurse affected caries prevalence in the experimental group. Finally, we restricted the primary analysis to only those participants who were enrolled and received their first examination and treatment in the 6 months prior to school shutdowns due to COVID-19 to ensure that all analyzed participants had the same elapsed time between observations. Statistical significance was determined at *P* < .05. Analysis was conducted in Stata version 18 (StataCorp) and R version 4.2.3 (R Foundation).

## Results

A total of 7418 participants were enrolled across 48 schools (mean [SD] age, 7.58 [1.90] years; 4006 [54.0%] female; 125 [1.7%] Asian, 1246 [16.8%] Black, 3648 [49.2%] Hispanic, 153 [2.1%] White, 114 [1.5%] multiple races or ethnicities, 90 [1.2%] other [unspecified], 2042 [27.5%] unreported) (Figure; Table 1). After randomization, there were 3739 participants (50.4%) in the experimental group (SDF) and 3679 (49.6%) in the active control group (sealant and ART). There were 4100 participants (55.5%) who completed at least 1 follow-up observation: 2063 (50.32%) in the experimental group and 2037 (49.68%) in the active control. The mean (SD) study observation time was 3.7 (0.32) years. The overall prevalence of baseline untreated caries was 26.7%, or 27.2% (95% CI, 25.7-28.6) for the experimental group and 26.2% (95% CI, 24.8-27.6) for the active control group. Within the SDF arm, 764 of 3735 baseline participants (20.5%) and 1154 of 8509 individual participant encounters (13.5%) were treated by registered nurses.

**Figure.**
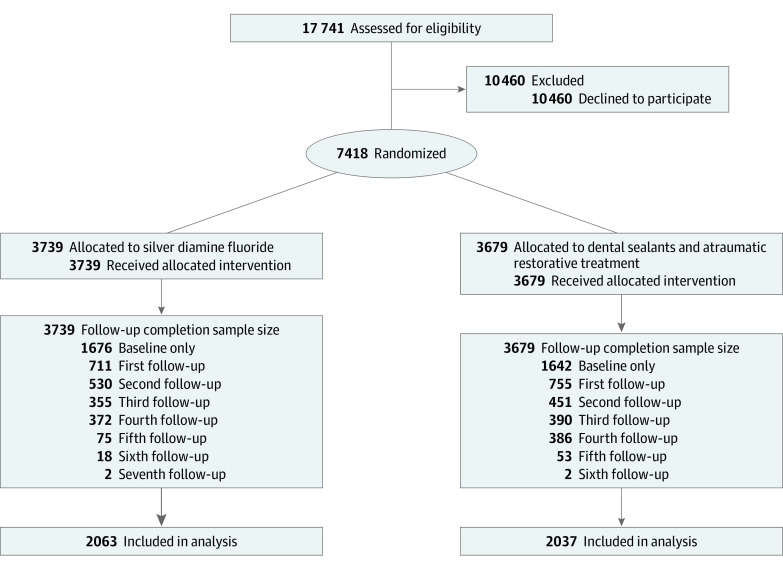
CONSORT Flow Diagram The average time elapsed between observations across all participants was as follows. First follow-up, 507 days; second, 300 days; third, 195 days; fourth, 169 days; fifth, 171 days; sixth, 170 days; seventh: 159 days. Any study dropout was considered and logged as lost to follow-up. No other reasons were documented, as the child most likely left the school.

**Table 1.** Sample Demographic Characteristics Overall and by Treatment

Characteristic	No. (%)		
	Overall	Experimental^a^	Active control^b^
Enrolled participants	7418 (100.0)	3739 (50.4)	3679 (49.6)
Baseline decay	1980 (26.7)	1016 (27.2)	964 (26.2)
Sex			
Female	4006 (54.0)	1954 (52.3)	2052 (55.8)
Male	3412 (46.0)	1785 (47.7)	1627 (44.2)
Race and ethnicity^c^			
Asian	125 (1.7)	88 (2.4)	37 (1.0)
Black	1246 (16.8)	650 (17.4)	596 (16.2)
Hispanic	3648 (49.2)	1766 (47.2)	1882 (51.2)
White	153 (2.1)	86 (2.3)	67 (1.8)
Multiple	114 (1.5)	67 (1.8)	47 (1.3)
Other^d^	90 (1.2)	56 (1.5)	34 (0.9)
Unreported	2042 (27.5)	1026 (27.4)	1016 (27.6)
Age at baseline, mean (SD), y	7.6 (1.9)	7.5 (1.9)	7.6 (1.9)
Retained participants	4100 (100.0)	2063 (50.3)	2037 (49.7)
Baseline decay	1140 (27.8)	584 (28.3)	556 (27.3)
Sex			
Female	2228 (54.3)	1088 (52.7)	1140 (56.0)
Male	1872 (45.7)	975 (47.3)	897 (44.0)
Race and ethnicity^c^			
Asian	78 (1.9)	59 (2.9)	19 (0.9)
Black	794 (19.5)	416 (20.2)	378 (18.6)
Hispanic	2329 (57.1)	1155 (56.0)	1174 (57.6)
White	86 (2.1)	56 (2.7)	30 (1.5)
Multiple	62 (1.5)	40 (1.9)	22 (1.1)
Other^d^	69 (1.7)	41 (2.0)	28 (1.4)
Unreported	682 (16.6)	296 (14.4)	386 (19.0)
Age at baseline, mean (SD), y	6.9 (1.6)	6.9 (1.6)	7.0 (1.6)

^a^ Experimental refers to treatment with silver diamine fluoride.

^b^ Active control refers to dental sealant and atraumatic restorative treatment.

^c^ Race and ethnicity data were collected via self-report using categories derived from the New York City Department of Education to support future assessment of effects within sociodemographic groups.

^d^ Unspecified.

The prevalence of participants with no new caries or fillings at each observation (Table 2) was similar in both groups, with differences in prevalence ranging from −0.001 to 0.031 across study observations. Bootstrapped confidence intervals were below the noninferiority margin. For mixed-model analyses of caries prevalence over time (Table 3), the interaction effect between time and treatment was not significant, indicating that noninferiority should be assessed marginally. Across both groups, the odds of untreated decay significantly decreased by approximately 21% at each observational visit (OR, 0.79, 95% CI, 0.75-0.83). Comparing the active control to the experimental treatment after adjusting for confounders, the OR was 0.94 (95% CI, 0.80-1.11; 90% CI, 0.82-1.08). The confidence interval for the marginal effect was outside the estimated OR δ.

**Table 2.** Prevalence of Participants Without New Caries or New Fillings at Each Observation^a^

Observation	Duration, d^b^	Prevalence		Difference (95% CI)
		Active control^c^	Experimental^d^	
2nd	507	0.67	0.64	0.03 (−0.00 to 0.07)
3rd	300	0.69	0.69	−0.00 (−0.04 to 0.04)
4th	195	0.7	0.7	0.00 (−0.05 to 0.05)
5th	169	0.76	0.75	0.01 (−0.03 to 0.06)

^a^ Any single instance of decay or new fillings not previously observed was considered treatment failure.

^b^ Duration was days between observations.

^c^ Active control refers to dental sealant and atraumatic restorative treatment.

^d^ Experimental refers to treatment with silver diamine fluoride.

**Table 3.** Longitudinal Caries Prevalence and Effect of Sealants and Atraumatic Restorative Treatment Compared With Silver Diamine Fluoride for Untreated Decay on Any Dentition

Variable	Odds ratio (SE)	95% CI	*P* value
Observational period	0.79 (0.02)	0.75-0.83	<.001
Active control vs experimental^a^	0.94 (0.08)	0.80-1.11	.47
Baseline decay	82.75 (9.72)	65.74-104.17	<.001
Previous care	0.76 (0.12)	0.56-1.02	.07
Sex, males	1.06 (0.09)	0.90-1.26	.47
Race and ethnicity^b^			
Asian	0.80 (0.24)	0.44-1.45	.47
Black	1.06 (0.12)	0.84-1.31	.61
White	0.66 (0.19)	0.37-1.15	.14
Multiple	0.87 (0.33)	0.42-1.81	.71
Other^c^	2.10 (0.64)	1.16-3.81	.01
Unreported	0.96 (0.12)	0.76-1.22	.75

^a^ Active control refers to dental sealant and atraumatic restorative treatment and experimental to treatment with silver diamine fluoride.

^b^ Race and ethnicity data were collected via self-report using categories derived from the New York City Department of Education to support future assessment of effects within sociodemographic groups.

^c^ Unspecified.

For newly observed caries across the full study duration (Table 4), the crude incidence rate in the experimental group was 10.2 caries per 1000 tooth-years. The rate in the active control was 9.8 caries per 1000 tooth-years, for a rate ratio of 1.05 (95% CI, 0.97-1.12) and a preventive fraction of 0.023. From adjusted models for longitudinal caries incidence (eTable 1 in Supplement 2), the overall risk rate over time reduced (incidence rate ratio [IRR], 0.83; 95% CI, 0.81-0.85) with each observation. The risk comparing participants in the dental sealants with ART group to those in the SDF group was 0.92 (95% CI, 0.83-1.04). There were no significant interactions between treatment and time and treatment and baseline decay status.

**Table 4.** Incidence Rate of Dental Caries for Experimental and Active Control^a^

Variable	Experimental^b^	Active control^c^	Total	Incidence rate difference (95% CI)	Incidence rate ratio (95% CI)
Caries, No.	1625	1433	3058	0.000 (−0.000 to 0.001)	1.046 (0.973 to 1.123)
Tooth-years, No.	157 979	145 653	303 632		
Incidence rate	0.010	0.010	0.010		

^a^ Each condition also received fluoride varnish.

^b^ Experimental refers to treatment with silver diamine fluoride.

^c^ Active control refers to dental sealant and atraumatic restorative treatment.

In supplementary analyses, the hazard ratio comparing the active control to experimental for time to first observed carious lesion was 0.91 (95% CI, −0.823 to 1.08). There were no significant differences in caries prevalence in children treated with SDF by registered nurses compared to dental hygienists (OR, 0.89; 95% CI, 0.67-1.19). For the restricted subsample, 4718 CariedAway participants were enrolled and treated in the 6 months prior to school closures due to COVID-19, 2998 of whom were viable for follow-up after pandemic restrictions were lifted (eFigure in Supplement 2). At the completion of the trial, follow-up data were obtained for 1831 participants (eTable 2 in Supplement 2). Results for longitudinal analyses for caries prevalence (eTable 3 in Supplement 2) and incidence (eTable 4 in Supplement 2) were similar to that of full-sample analyses.

## Discussion

School sealant programs have demonstrated effectiveness in reducing the risk of dental caries,^27^ yet are underused due to the burdensome costs of care.^9^ Many children subsequently continue to live with untreated disease, which can lead to systemic infection and negatively affect child development.^28^ In this randomized clinical trial of primary school-aged children, application of SDF with fluoride varnish was noninferior compared to dental sealants, fluoride varnish, and ART in the longitudinal prevalence of caries when used in a school program. We conclude that SDF is an effective alternative for community-based prevention that may help address these existing barriers.

Although SDF is primarily used as a caries-arresting agent, it is also effective in the prevention of new caries.^29,30^ There is a reduced risk of new caries on surrounding sound dentition when existing lesions are treated,^31^ and SDF is more effective than fluoride varnish in preventing new caries in early childhood.^32^ However, prior short-term comparative assessments of SDF have yielded conflicting results on its superiority relative to glass ionomer sealants and atraumatic restorations.^33,34^ These previous trials were also restricted to either 12 or 24 months of observation, and little long-term evidence exists.^10,22^

Approximately 1 in 4 of children participating in CariedAway had untreated caries at baseline (1 in 3 for the COVID-19 sample), and 11% had preexisting sealants. Following treatment, the overall odds of dental caries decreased by approximately 20% in both study arms. The risk of incident dental caries was nearly identical in both treatment groups, resulting in a very small preventive fraction between the included interventions. Similarly, the data indicate no significant differences across treatment in the risk of first caries eruption or when modeling the total number of new dental caries experienced overall, nor is there sufficient evidence to indicate whether there are differences in treatment effect over time or based on the presence of disease at baseline. Dental sealants have an estimated 50% preventive fraction for caries compared to placebo, with research estimating the prevention of more than 3 million cavities attributable to sealants.^35^ The similarity in observed incidence from CariedAway may support a similar conclusion for the application of SDF.

In addition to clinical effectiveness, the simplicity and financial implications of a school-based SDF program can result in considerable cost savings to the public. A review of existing SDF treatment protocols identified application times as low as 10 seconds per tooth,^12^ suggesting that more children can be treated in less time. Use of SDF as a caries management strategy also reduces Medicaid program expenditures,^36^ is the most cost-effective option in populations with a high risk of dental caries,^37^ and is more cost-efficient compared to ART,^13^ although potential restrictions from Medicaid reimbursement may persist.^36^

In 2022, the American Medical Association approved a category III *Current Procedural Terminology *code authorizing nondental health care professionals to administer SDF, and research indicates that treatment of early childhood caries using SDF by physicians in primary care settings is both feasible and acceptable.^38^ Similarly, the American Academy of Pediatric Dentistry published guidance on physician use of SDF for caries management,^39^ and surveys of pediatricians by the American Academy of Pediatricians reveal an interest in and recognized need for SDF.^40^ More than one-fourth of participants in the SDF arm of the CariedAway trial were treated by registered nurses, and our results for incident caries over 4 years corroborate other findings on the effectiveness of nurses in providing SDF.^41^ School-based caries prevention may have greater student participation when school nurses partner with hygienists in the delivery of care,^42^ and our results empower nurses as primary agents in caries prevention. With more than 132 000 school nurses estimated to be currently in the US^43^ and given their growing involvement in oral health promotion and prevention,^44^ these findings can expand the scope of practice for both school nurses and family practices.

While the American Dental Association^45^ and the American Academy of Pediatric Dentistry^46^ include SDF in their clinical recommendations for caries management, known complications with SDF application include potential oral soft tissue irritation, temporary staining of the oral mucosa, and permanent staining of porous tooth structure.^29^ Despite thousands of SDF applications in CariedAway, we encountered no adverse events and received only 1 complaint regarding staining, which pertained to superficial skin staining from accidental spillage that was mistaken for bruising. Separate findings from CariedAway did not indicate a negative impact of SDF therapy on oral health-related quality of life, which included measures for aesthetic perceptions of the oral cavity.^47^ Other research concludes that a high proportion of parents of children treated with SDF remain satisfied with their child’s dental appearance,^48,49^ that aesthetic concerns are mitigated with posterior application,^50^ and that no differences were found in adverse events or aesthetic perceptions when comparing children treated with SDF vs sealant and ART.^51^

### Limitations

As a pragmatic trial, there are concerns regarding subject attrition and any bias from external care. Our analysis used all available observations for study participants, considered a subset of participants that had equal rates of follow-up due to COVID-19, and identified any treated dentition by clinicians outside the CariedAway program. Additionally, the presented findings assessed caries prevalence inclusive of both children who did and did not begin the study with active untreated decay, which has, to our knowledge, not been reported previously, as those with untreated caries at baseline may have a higher risk of subsequent disease development. We also included multiple assessments of prevention, including any incidence of decay, overall prevalence, time to first eruption, and estimates at both the tooth and person levels. While attrition is a clear weakness, the pragmatic nature of the trial reflects the real-world experience of a school-based model that uses SDF for long-term caries management.

## Conclusions

Untreated dental caries has maintained a 30-year position at the top of the global disease prevalence lists.^52^ Results from the CariedAway randomized clinical trial demonstrate the longitudinal effectiveness of SDF when used in school-based caries prevention and can be used by clinicians, practices, and communities in the global pursuit of oral health equity.

## Notes

Supplement 1. Trial protocol

Supplement 2. eTable 1. Longitudinal caries incidence and the effect of sealants with ART compared to silver diamine fluoride, full sample eFigure. CONSORT diagram, COVID-19 sample eTable 2. Sample demographics overall and by treatment, COVID-19 sample eTable 3. Longitudinal caries prevalence and the effect of sealants with ART compared to silver diamine fluoride, COVID-19 sample eTable 4. Longitudinal caries incidence and the effect of sealants with ART compared to silver diamine fluoride, COVID-19 sample

Supplement 3. Data sharing statement
