# Supplementary material for: Noninferiority of Silver Diamine Fluoride vs Sealants for Reducing Dental Caries Prevalence and Incidence: A Randomized Clinical Trial
Source: JAMA Pediatr. 2024 Mar 4;178(4):354–61. doi: 10.1001/jamapediatrics.2023.6770 (PMC10913007; doi:10.1001/jamapediatrics.2023.6770)
Supplement: Supplement 1. — Trial protocol [file jamapediatr-e236770-s001.pdf]

PCORI RESEARCH PLAN

RESEARCH STRATEGY

A. Background

Introduction

The immediate goal is to determine the effectiveness of two, school-based, caries prevention programs (**RQ-1**): a "simple" regimen consisting of silver-diamine-fluoride + fluoride varnish, and a "complex" regimen consisting of traditional sealants + therapeutic sealants + fluoride varnish. We will test the hypothesis that school-based "simple" caries prevention is as effective as school-based "complex" caries arrest and prevention. We will use a school-based cluster randomized controlled trial design to test this hypothesis (**RQ-2**). The population focus is the low-income Hispanic/Latino community with limited access to oral health care **(RQ-3).** More specifically, we will focus on high-risk, school age children from this community **(RQ-4)**. We selected two evidence-based and school-based prevention protocols because they directly increase care access and overcome multiple barriers to care **(RQ-5).** We will assess the following outcomes, based on community meetings and surveys: caries, quality of life (including toothaches), and school performance **(RQ-6).**

Based on 3 years of conversations, surveys, and guidance from parents and stakeholders **(PC-1)**, we: selected school-based caries prevention and focused on NYC elementary schools that serve the NY States' highest need, low-income, Hispanic/Latino community [1-3] (**PC-2**). Parent and stakeholder partners recommended that we track the following patient-centered outcomes: untreated cavities, quality of life, and school performance (**PC-3**). Parent and stakeholder partners participated in planning, designing, and piloting the proposed study. These partners will participate in the proposed study's conduct, dissemination, and implementation to NY City's some 1,200 elementary schools serving some 80,000 low income minority children **(PC-4)**. Beyond NY City, and beyond academic publications, our stakeholder partners include United Concordia and Univision. United Concordia is a national dental insurer, including insuring the U.S. military. Univision is the 4th largest U.S. media network whose audience is the Hispanic/Latino community. Working with both we will increase the probability for a national impact.

In sum, the study rationale and design, while based on local input and needs, reflects national evidence gaps and needs. These include PCORI Research Prioritization Topic Brief 5[4], care access barriers [5,6], demographic needs [7-10], dentist's underuse of prevention [11-13], and the absence of prevention protocols with demonstrated effectiveness.[14-16] Our partnerships, in addition to our team's prior experience, brings us confidence that our approach meets community needs and circumstances, employs relevant assessments, and will allow us to successfully disseminate and implement the proposed program.

Critical Evidence Gaps (RQ-1)

Criterion 1. Potential for the study to fill critical gaps in evidence.

This project addresses the following critical evidence gaps: 1) The global and national burden of children's caries; 2) the burden of disease in New York City, particularly among Hispanic/Latino children from low-income families; 3) the social burden of disease, particularly academic performance and psycho-social development; 4) Barriers to office-based care; 5) Economics support treatment; 6) Treatment is less effective than prevention; 7) Effective prevention is available; 8) Prevention is underused; 9) Large variation in care, without outcome measures of effectiveness; 10) No systematic reviews of prevention effectiveness; 11) Standard of care is not established.

The proposed school-based, cluster, randomized controlled trial, comparing two evidence-based caries prevention protocols, will address and potentially obviate all of the identified gaps.

Gap #1: Global and National Disease Burden: Caries is the globe's and U.S.'s most prevalent, uncontrolled, preventable infection [17]. Caries prevention is a PCORI Research Prioritization Topic [4], and untreated caries is number 19 on the Institute of Medicine's top 100 questions for comparative effectiveness studies [18]. More than 50% of U.S. elementary school-age children have experienced caries, and more than 20% have untreated caries. For Hispanic/Latino, low-income children caries experience is over 70% and the untreated caries is over 30%[7-9]. Even more problematic,

children with untreated caries have an incidence of sepsis ranging from 5% to 10% [19]. In rare instances, untreated decay can lead to serious systemic infections and even death [20].

Gap #2: Disease Burden in New York City (RQ-3, RQ-4). Children's oral health needs, and particularly Hispanic/Latino children from low income families, exceed U.S. averages[5,10,21]. Current estimates indicate that 38% have untreated caries, 2/3 do not have sealants, and all have difficulty accessing care.

Gap #3: Social Burden: Untreated caries is associated with poor academic performance and poor psycho-social development. Children with untreated caries and associated toothaches have more school absences, cannot pay attention in school, do not keep up with their peers academically, and have lower standardized test scores[22-26]. Poor oral health is also associated with inhibited growth and development, psycho-social vulnerability, lower well-being and self-efficacy, and reduced locus of control[27-31]. Numerous explanatory theories[32,33], life-course approaches[34], frameworks for action[35], and analytic methods[36] are proposed for assessing and improving children's oral health equity. However, we were unable to identify any reports of prospective causal studies that validate these associations. The prospective study proposed here will test the effect of oral health on school performance and quality of life.

Gap #4: Patient Barriers to Office-based Care. Office-based care, and particularly surgical care, presents multiple access barriers for patients including, in decreasing order of patient importance: cost, fear, geographic location/travel, time, knowledge, culture and literacy [37-40]. These concerns were mirrored in parent/stakeholder discussions (see below).

Gap #5: Economics Support Overuse of Treatment[6]. From 2003-2009, fewer than 15% of children who accessed dental care received topical fluoride or sealants[13], and in 2013, fewer than 16% of 6-9 year olds received sealants[12]. Further, fewer than 40% of U.S. dentists provide sealants[11]. In NYC, for children 6-12, 65% of children have never had sealants, and for children who have seen a dentist 60% were not given sealants [10].

Consequently, from 1990 to 2010: while the number of practicing U.S. dentists and hygienists increased by 30% and 60%, respectively[41], and Medicaid spending for children's oral health care increased 7-fold, from $1b/year to $7b/year[42], children's caries experience continued to increase [43]. The burden was particularly severe for minority, low-income children, and particularly Hispanic/Latino children[7-10].

Gap #6: Surgical Care ("drilling and filling") is Less Effective than Preventive Care. Surgical care uses local anesthesia, followed by surgical removal of decay and tooth structure to place a filling[44]. These fillings are not permanent: they have a limited life span of ~10 years and then require replacement by a larger restoration[45-47]. The net result is that fillings, once placed, must be repeated with more extensive and expensive restorations[48,49]. Prevention, and particularly, therapeutic sealants provided in the complex arm proposed here, restores teeth without surgical intervention, is as long lasting, and has fewer adverse events than classical restorative care[50,51]. From these perspectives, the current surgical care model is a clinical example of the over-use of ineffective therapy, and the under-use of effective prevention[6,52-56]. We therefore propose a definitive study examining the effectiveness of community-based prevention.[6,57]

Gap #7: Effective Caries Prevention is Available. Systematic reviews verify the efficacy for numerous caries preventing agents (**Table 1**) [54]. The identified systematic reviews examined trials of individual agents. Our work, in contrast, demonstrates that, when used in combination, and delivered by dental hygienists in schools, complex preventive care can be effective in reducing the prevalence/incidence of caries [72,73]

Table 1. Systematic Reviews of Caries Preventive Agents (Summary)[54] (SR-1)

Agent	Frequency	Est. % Efficacy
**1. Water Fluoridation**	Continuous	20-40[1]
**2. Fluoride toothpaste**	2X/day	25[2]
**3. Fluoride varnish**	More than 2X/year	40 [3]
**4. Traditional Sealant**	1X /pits & fissures	80 [4]
**5. Therapeutic Sealant***	1x/ caries	80 [5]
**6. Silver-diamine-fluoride**	2X/year	80 [6]

References: 1. [58-61]; 2. [62,63]; 3. [64]; 4. [65-67]; 5. [50,51,65,68] ; 6. [69-71]. * Seal caries to prevent further tooth destruction. Requires no anesthesia or drilling. Also called: Interim Therapeutic, Atraumatic, or Temporary Restorations, Minimal Intervention Dentistry. ** CHW: Community health worker

Gap #8: Prevention is Cost Effective, but is Underused. Systematic reviews with economic assessments indicate that: 1) caries prevention is cost-effective[74-76]; and 2) investment in prevention outweighs investment in fillings[50,76-79]. Further, when compared to traditional fillings: 3) therapeutic sealants require no excision of tooth structure and have significantly fewer adverse events such as acute pain and endodontic involvement [50,51].

Gap #9: Clinical Variation without Outcome Measures - "Know-what" and "Know-how"[80]. While at least 13 reports [81], and multiple federal agencies recommend school-based caries prevention[82-84], these same reports recognize the "know-what" "know-how" gap. That is, the gap between knowing what works in clinical trials (efficacy) and knowing how to implement them in a community setting to demonstrate effectiveness.[38,48,49,85,86] For example, we identified large variations in community-based care based in: 1) two national surveys[14,15]; 2) our systematic assessment[54]; 3) our key informant interviews[16]; and 4) our insurance analysis[6]. This variation includes frequency of care, intensity of care, type of care, and target age for intervention. As well, while we identified multiple process measures, we were not able to identify outcome assessments related to caries prevention. Supporting our assessment are recent systematic reviews[87,88].

Gap #10: Absence of Systematic Reviews (SR-1). To identify prior work on place-based caries prevention we queried PubMed using the following conceptual search strategy:

(dental AND (caries OR cavities OR decay)) AND prevention AND (school OR place) AND (effective OR effectiveness)

We included synonyms for all terms and MeSH headings (N=717), then limited the search to systematic reviews (N=63) and clinical trials (N=503). We then examined the titles and abstracts. Our inclusion criteria were effectiveness trials with concurrent implementation of (i) fluoride varnish, (ii) sealants on all pits and fissures, (iii) therapeutic sealants, and (iv) provision of toothbrush and toothpaste. We identified no effectiveness studies that met these inclusion criteria. We then re-examined the titles and abstracts for trials that only provided: (i) fluoride varnish and (ii) sealants on all pits and fissures. We again identified no studies. The preponderance of studies examined efficacy of single interventions. When multiple interventions were used (e.g.: fluoride varnish + sealants) the sealants were not placed on all pits and fissures. Parallel searches of the Cochrane Library and Web of Science did not identify any prior work.

However, a review of reference lists identified the 30 year-old National Preventive Dentistry Demonstration Program [89,90]. This 6-arm, 10-site, national, elementary school-based, cohort study, did compare multiple preventive agents. The study found that dental health lessons, brushing and flossing, fluoride tablets, fluoride mouth rinse, fluoride toothpaste, and fluoride gels were not effective in reducing caries, even when used together, but that sealants were effective [91-93].

Not surprisingly, there are effective new agents (e.g: fluoride varnish, fluoride releasing glass ionomer sealants, silver-diamine fluoride), and new methods (therapeutic sealants) that were not available 30 years ago. As well, the children in NYC, low-income, high-minority schools have substantially greater oral health needs than those in the National Preventive Dentistry Demonstration Program.

Gap #11: Absence of a Care Standard[94]. The standard of care is generally defined by the Frye rule, meaning acceptance among local experts. However, that rule was superseded by the Supreme Court's Daubert ruling in 1993 requiring only sound scientific evidence be admitted in court [95], and followed this ruling with a reference manual [96]. Dental practices that do not provide preventive care with proven efficacy operate outside the standard of care.

Final Comment. To address these gaps we propose a cluster randomized controlled trial, in NYC elementary schools serving predominantly Hispanic/Latino children from low income homes, to compare two evidence-based prevention protocols. Current data suggests that both protocols could address and obviate all of the identified gaps.

B. Significance

Criterion 2. Potential for Study Findings to be Adopted into Clinical Practice and Improve Care Delivery.

From a health, business, ethics, and legal view, we've made the case for caries prevention, and in particular universal PreK-to-grade 8 caries prevention.[6,57] The proposed work will further support this.

In NY City, NYU Dental and NYU Nursing in collaboration with the NY City Departments of Education and Health and Mental Hygiene will use the results of this program to potentially provide caries prevention to 800,000 underserved NY City school children. State wide, the NY chief dental officer, Medicaid dental officer, and third-party Medicaid provider are all interested in outcomes that will inform their programs. Finally, a national dental insurer has similar interests.

Criterion 5: Patient-centeredness.

The proposed program evolves from discussions with, and surveys of, Bronx Latino/Hispanic low-income parents of school age children beginning in 2013. The proposal incorporates multiple patient-centered requests including: care locations (schools), cost of care (free), simultaneous application of multiple preventive agents (simple or complex prevention), potential color change (silver diamine fluoride on posterior teeth only), improved health (arrest cavities), better academic performance and quality of life (including toothaches)

The parents expressed concerns about office-based dental care, a preference for school-based care, and a focus on prevention of cavities, as well as school performance and quality of life. These concerns and preferences were similar to those of school parent coordinators.

These discussions led to an assessment of available individual preventive measures, and grouping of preventive measures into "simple" and "complex". Simple takes ~1/4 the time and has ~1/4 the cost of complex prevention. Therefore, simple increases access to care 4-fold, when compared to complex.

All of the medicaments of both simple and complex are currently available, have evidence of efficacy. However, they have not been compared individually or in combination, so we do not have data on comparative effectiveness. Based on first principles, we posit that the two methods are equally effective.

Improving Evidence for Patient and Stakeholder Decisions

Care Location Decisions (RQ-3,-4, -5; PC-1, -3). Beginning in 2013, in multiple discussions with parents of school age children (see section E. Engagement Plan), parent coordinators, school nurses, and school administrators, there was general concurrence that there are three choices for dental care: office-based surgical care (community health center, dental office, or van where available), no care, or emergency room care. None of these choices are optimal. Parents voiced vigorous concerns about the cost of care, fear of dentists, and time away from school/work for their children. These concerns parallel national surveys.[37-40]

Based on our experience we queried parents about school-based prevention as an alternative, and found interest. To verify this interest, we carried out two surveys: a survey of parent coordinators across NY City elementary schools and a survey of parents of children and teachers in two Bronx elementary schools. We found that school-based care was overwhelmingly preferred over office-based care (**Table 2**). These surveys also identified school absences due to toothaches as a particular concern. This parallels a national survey in which 31% (95% CI ± 2.7%) of parents said their children age 6-12 missed school due to dental problems[26].

Based on these results and our conversations with parent partners, the proposed program offers a fourth and the preferred care choice: school-based caries prevention. These results and conversations also led us to propose the assessment of: 1) caries arrest, the upstream antecedent to toothaches; 2) caries prevention; and 3) school absence, performance, and oral health related quality of life, the downstream consequences of effective/ineffective caries prevention.

Table 2. Survey of Partner Concerns and Preferences

	**Stakeholders**		
	**Bronx Parents**	**Bronx Teachers**	**NYC Parent coordinator**
Survey response rate	43% (310/724)	75% (76/102)	47% (155/331)
Children with school absence for toothache	17%	27%	36%
Prefer school-based to office-based care	76%	97%	72%

Importantly, our conversations with parent partners also indicated that they want care that is independent of a family's ability to pay. This clearly affects sustainability (see below).

Better Decision Making (RQ-3). From a behavioral perspective, school-based prevention is an easy decision[97,98]. For parents, school-based care overcomes 9 identified barriers to traditional dental office-based care, including: cost, fear, geographic location, travel, time, school/work absence, knowledge, culture and literacy [37-40]. Concretely, from a child's perspective, bringing care to children within their learning environment, and in their language, facilitates normative health communication literacy, knowledge and cooperative behavior, and reduces fear[57]. For parents, providing children with school-based preventive care, regardless of a parent's ability to pay, reduces costs and time. For the program proposed here, we will provide care to all children with informed consent, independently of a parent's ability to pay.

Quality and Health Concerns (RQ-5). In our conversations with parents, they routinely ask about how safe and how good school-based care is. Our work in Massachusetts [72] and Colorado [73] indicates that "complex" school-based caries prevention adheres to all 6 quality aims from the *Institute of Medicine* .[99] Care is safe, effective, patient-centered, timely, efficient, and equitable. Further, our work indicates that "complex" caries prevention improves oral health (**Figures 1-3).** Specifically, with each preventive visit there is a reduction in: untreated caries (**Figure 1**); acute infection (**Figure 2**); and new caries risk (**Figure 3**). Cumulatively, we found that over two years participants can expect >50% reduction in untreated caries [72,73]. We expect similar results with simple prevention.

Recent systematic reviews indicate that complex prevention is superior to classical fillings [50,51,79,100]. Conversely, at the other end of the clinical spectrum, there appears to be no benefit for caries screening and referral for care[101]. Consequently, without school-based prevention parents can expect their children to encounter increasing incidence and prevalence of caries and need for emergency care.

Figures 1-3. Preliminary data showing that school-based oral health care improves health outcomes in children at risk for untreated caries.

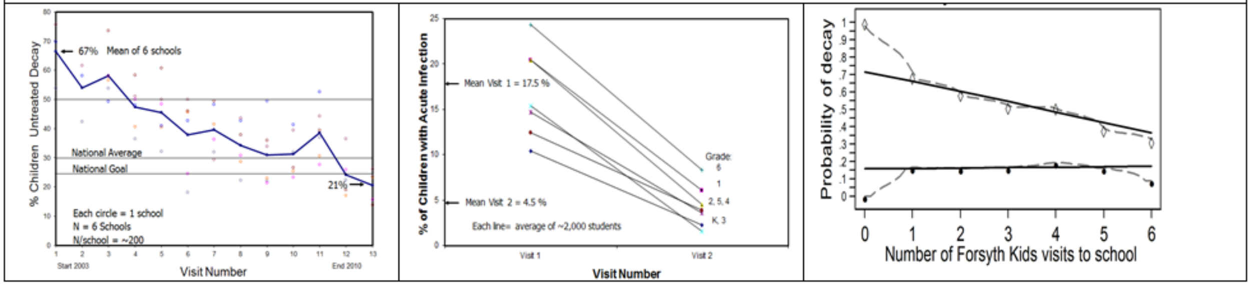

Sustainable Access to Care (PC-4). New York City is currently in the process of embedding school-based health care into all of its schools to increase sustainable access to care. This will provide policy drivers for sustainability. Economically, care delivery costs approximately ~$20 for "simple" prevention and ~$80 for "complex" prevention (these figures include supplies, salary, fringe benefits and overhead[6]). The current New York Medicaid encounter fees are $158.55 per visit. The difference between the cost and encounter fee ranges from $79 (for complex prevention) to $139 (for simple prevention). Consequently, the proposed program should be financially sustainable with either simple or complex care.

Clearly, however, Medicaid does not cover all children. In New York City, >60% of children are Medicaid participants. In our 2 pilot schools (see section E. Engagement Plan) 60% are Medicaid participants, and Medicaid covered 80% of submitted billing. This difference allowed us to provide free care to children without Medicaid.

We expect the identified figures to hold true in the future. While refined economic analysis is beyond the scope of this proposal, we've published some simple economic assessments that support the business case for school based, and more specifically, universal PreK-to-grade 8 caries prevention[6].

Local and National Dissemination (PC-4). We've argued for a universal Pre-K to grade 8 school-based caries prevention program [6,57]. We've based this on national Healthy People aims, on a business case, on the current best evidence, and on the ethics of health equity. We will continue to make this case.

In NY City, Dr. M. De Stefano, Director of School-Based Health Centers, Dental Clinics and Health Insurance at the Department of Education, asked the PI 3 years ago to implement school-based caries prevention in 1,600 NY City schools and provide care to some 800,000 children (see section E. Engagement Plan and Letter of Support). This proposal is a first step toward refining a program to do this.

To improve the potential for dissemination, given the workforce limitations of U.S. dentists (~150,000) and dental hygienists (~200,000), we are collaborating with NYU Nursing. There are approximately 3 million U.S. nurses. NYU Nursing has already taken a national leadership role, and won awards, for inter-professional oral health education and care. They coined the term HEENOT, expanding HEENT (head, ear, eyes, nose, throat) to include "O" for oral. If successful, the proposed "simple" program could be included in all U.S. nursing programs.

To improve the probability of local clinical dissemination and implementation, our parent partners include Latina mothers with school age children as a Co-I (Ms. N. Santiago), a Clinical Coordinator (Ms. B. Alicea), and a Lead School Coordinator (Ms. N. Lopez). We worked with all of these individuals in developing the current program and in our pilot

schools. Our local Stakeholder Partners are all Hispanic/Latinos and include: Bronx PS 140 principal (Mr. P. Cannon), Deputy Director for Health Administration at the Bronx non-profit, Children's Aid Society (Ms. A. Cruz), and the Administrative Director for Community Pediatric Programs at Montefiore Health Systems (Ms. P. Christian).

In addition to Dr. De Stefano, guiding us at the NYC Department of Education are the CEO, Office of School Health, Dr. R. Platt, and the Director of Oral Health Programs at the NYC Department of Health (Ms. R. Kalra).

The following individuals are Stakeholder Partners with state-wide responsibilities, and can facilitate state-wide implementation. They are: Dr. D. Richardson, the NY State Dental Director; Dr. G. Gostling, NY State Medicaid Dental Dirctor; Ms. S. Zelkind, CEO of Healthplex, the largest NY State dental insurer, and a third party Medicaid administrator.

Dr. W. Maas, Retired Asst. Surgeon General, and Dr. J. Bramson, the Chief Dental Officer at United Concordia will provide national perspectives as members of the Patient and Stakeholder Partners. Dr. Maas is currently a dental consultant for the Pew Trusts. Dr. Bramson oversees the third largest U.S. dental insurer. United Concordia administers TriCare which covers the U.S. military. If the proposed program is successful, they can advocate for wider dissemination both within their respective organizations and nationally.

Letters of support from these individuals are included in this application. The letters indicate their interest in the outcomes of the proposed work to assist them in designing and disseminating better caries prevention programs.

Dissemination Barriers. There are four significant barriers to implementation: state practice acts, reimbursement, workforce availability, and access to schools. We've previously pointed out that current practice acts and reimbursement rates that do not support or incent effective, evidence-based caries prevention programs violate the Supreme Court's Daubert ruling[6,57,94], and may place states at legal risk. This will await a legal challenge. Because of the limited number of dentists and hygienists, and the >10-fold greater number of nurses, there should be little demand shock to national implementation if simple prevention is as effective as complex prevention and nurses are used to deliver simple prevention. Finally, school access is dependent on the local principal. We are confident that if our work demonstrates that, following improved oral health, school performance increases, and school absence decreases, there will be an increasing demand for caries prevention programs.

Addressing Patient Questions and Outcomes (RQ-3, -5, -6; PC-1, -3)

Preventive Care Questions (RQ-3, -5; PC-1). In our discussions and survey of parent partners, and the acknowledged fear of dentists, and particularly shots and drilling, and their concerns about cost of care, we focused on upstream preventive interventions. We shared with them the data clinical efficacy from **Table 1.** Since NY City water has fluoride we focused on 2-6, and the National Guideline Clearinghouse[102]. The latter lists 4 guidelines supporting fluoride toothpaste, 10 guidelines supporting fluoride varnish, 6 guidelines supporting sealants, and 5 guidelines supporting therapeutic sealants. Silver diamine fluoride does not yet have guidelines.

In discussing options with parents, teachers, nurses and parent coordinators, one parent coordinator asked: "Since you in the school, why don't you just do all of them?" This discussion evolved to looking at what combination(s) might be most effective, cost effective, and efficient. A comparison of two protocols – "simple" and "complex" – evolved out of these discussions.

Silver Diamine Fluoride Questions (RQ-3, -6; PC-1, -3). We alerted parents about clinician's concerns about the color change following silver diamine fluoride application. (Silver diamine fluoride changes the color of frank caries from brown to black). However, they seemed to be more concerned with obtaining care than they were with color change.

To examine parent perceptions of color change we conducted a convenience sample survey of 120 parents (44 Hispanic/Latino parents) presenting for care at the NYU Dental Center or two community dental practices that treat low-income parents. The survey included photographs of anterior and posterior teeth before and after silver diamine fluoride treatment. We individually asked parents if the color change was unacceptable (score 1) to acceptable (score 4). For the posterior teeth 68% found the color change somewhat acceptable or acceptable, while 30% found it somewhat acceptable or acceptable on anterior teeth. Using a script, we then asked what their preference would be if the child exhibited different behaviors while getting fillings (e.g.: cooperative, upset, cried, kicked and screamed, needed sedation, needed general anesthesia). When behavior was considered, 60% of parents found anterior color change somewhat acceptable or acceptable. These results were independent of race/ethnicity, education, or income level. This suggests

that silver-diamine-fluoride would be an acceptable treatment for posterior teeth. Dr. R. Platt, CEO Office of School Health, NY City Departments of Education and the NY City IRB approved silver diamine fluoride for posterior teeth.

Outcomes of Interest (PC-1, -3). As articulated in the support letters from parent and stakeholder partners, we met repeatedly over the last 3 years to identify the needs, outcomes, and measures of import. Not surprisingly, the important outcome measures were similar to those posed in the PCORI Research Prioritization Topic Brief 5 [4]. They include access to arrest of caries, reduction in cavities, reduced school absence, improved school performance, and quality of life.

C. Patient Population

INTRODUCTION (RQ-3, -4, -5; PC-2)

The long-term focus of this program is on improving oral health equity, particularly for at-risk minority children from low-income families. The mid-term focus of this program is on the 800,000 at-risk children attending some 752 Title 1 New York City elementary schools that serve children from families with incomes $\leq$ 138% of the federal poverty level. The immediate focus of this program is on the highest need, low-income, Hispanic/Latino elementary school children, living in the Bronx and surrounding areas, with the lowest care access in New York City.

Study Population. Hispanic/Latino Low-income populations. We selected the Bronx and Northern Manhattan, which is the lowest-income county in NY state, with a median household income of $34.3k and the highest Hispanic/Latino population in the NY state (55.1% of 1.46m people)[1].

Specific Population (RQ-3) and Participant Subgroups (RQ-4). Within the Hispanic/Latino, low-income community we are focusing on children. Specifically, we are focusing on elementary school children from Hispanic/Latino, low-income families, living in the Bronx. Other surrounding boroughs with low-income, Hispanic/Latino communities are also applicable.

Source (PC-2). According to the NY City Department of Education data: 1) There are 99 Bronx elementary schools (grades PK to 8) with a preponderance of Hispanic/Latino children (average 69% per school; range: 51% to 90%). 2) The total enrollment in these schools is 53,089 (average 596 per school; range: 84 to 1705). 3) Almost 95% of these students come from families at or below 138% of the federal poverty level, and 60% of the children are current Medicaid participants.

In addition to the 99 Bronx elementary schools, there are 68 elementary schools serving 34,762 children with similar demographics in Manhattan, Queens, Brooklyn, and Staten Island[2]. More broadly, of the approximately 1,800 NY City public schools serving 1.1m children, almost 1,000 schools serve some 800,000 children from low-income families (<138% of the federal poverty level)[103].

School Solicitation (PC-2). NY City Department of Education identified and solicited principals in 30 elementary schools for participation in YR 01, and will solicit and identify up to an additional 30 for YR 02. Participating schools will be randomized to receive either simple or complex prevention. All children in a given school, with informed consent, will receive the same preventive care twice per year and followed through the duration of the study. Each year, new students enrolled in the school will be solicited and eligible for participation.

School Population. We estimate that the maximum enrollment of 60 schools serve approximately 35,393 students (60 schools x 590 students/school). All children with informed consent will be included in the study.

Included and Excluded Children. All children attending a participating school, with informed consent, will receive care, independent of race, ethnicity, sex, or ability to pay. Children with informed consent can join at any time. Only children without informed consent will be excluded.

Parent Coordinators. Each New York City school has a Parent Coordinator. Parent coordinators are bi-lingual parents, from the community, hired by the city, who report to the principal, and assist with all school-parent issues. For this program we will hire 3 similarly experienced individuals to be school liaisons to oversee the informed consent process.

Recruitment/Informed Consent Plan. For informed consent we will leverage guidelines from the National Quality Forum and Agency for Healthcare Research and Quality [104-106], and implement refinements, based on guidance from our parent stakeholders and parent coordinators. As done in our other school programs, parent coordinators on the research team will collaborate with parent coordinators at each school. Coordination will include the timing and process

of distribution and collection of informed consent, as well as the timing, location, and process of preventive care delivery within a school. Typically, this involves multiple conversations with the school administration to avoid vacation, testing, and other conflicts.

To facilitate accurate informed consent and student tracking the NY City Department of Education (NYC DOE) agreed to provide electronic student rosters for each school. The roster includes a child's name, address, date of birth, sex, parent's names, phone numbers, emails and unique identifier. This will facilitate personalizing printed informed consent with the school and child's name and tracking students longitudinally. The NYC DOE has translation services, and agreed to translate all forms into the language of the parent.

Working with parent stakeholder / parent coordinators in the two pilot schools, we created a parental FAQ, and a cover letter from the principal. This letter, FAQ, consent and health history were printed two-sided on 17'x11' paper and folded in half with serration down the center, forming a 4-page 8.5"x11" "book". This facilitated parents keeping the first page (principal letter and FAQ), and sending the second page (informed consent/health history) back to the school.

In the pilot schools, starting in the middle of the school year, the informed consent rate was 33%. Typically, we start at the beginning of the school year and expect a starting participation rate of 40%, with increasing participation over time. We expect the same to occur in the proposed program. According to the NY City Department of Education, informed consent tops out at just over 80% for schools with long standing school-based health centers. Thus we expect informed consent to be 40% by YR 01 in schools that start in YR 01, and above 50% in YR 02-05. We will enroll approximately 10,573 children within enrolled schools. Assuming a maximum school-level enrollment of 60 schools, this equals an average of 176 subjects per school. However, we note that should the average school enrollment be higher, full study enrollment may be achieved with less than 60 participating schools (**Table 3**). **Table 4** provides the expected demographics, and confirms large Hispanic/Latino population from thirty randomly sampled schools meeting study inclusion criteria.

Table 3. Recruitment Plan

Total number of study participants expected to be screened	10,573
Of those screened, total number of study participants expected to be eligible	0
Target sample size (use same number stated in milestones)	10,573

Table 4. 60 School Demographics

Race	Male (N)	Female (N)	Total (N)
American Indian/Alaska Native	0	0	0
Asian	615	591	1205
Black/African American	4222	4057	8279
Hawaiian/Pacific Islander	0	0	0
White	521	501	1022
Multi-race	180	173	353
**Ethnicity**	**Male (N)**	**Female (N)**	**Total (N)**
Hispanic (Latino/Latina)	12511	12023	24534
Non-Hispanic	5717	5494	10859

School Solicitation. To identify school participants, any primary school in New York City that had a Hispanic/Latino/black student population ≥50% and had at least 80% of the student population receiving free or reduced lunch ("Title 1 schools") is eligible to participate. Every school meeting the above criteria was solicited to participate. Preliminary demographic focus is in the Bronx. Schools with an existing school-based dental health program (e.g., sealant programs) were excluded from the study solicitation. School principals were sent letters describing the study protocol and interventions, and any interested principal opted into the program. A list of schools requesting participation is included.

Participation Barriers. For school-based care there is only one barrier – informed consent. The previous paragraphs address this. Given our prior experience, and national data, we expect to achieve between 40% and 50% informed consent. However, to insure that we are at the higher end, the research team will include parent coordinators.

Final Comment. Two points of comparison are relevant here. First, as indicated in the Background, office-based care presents 9 access barriers[5,40], and second, office-based care provides preventive care to less than 20% of the population[11-13]. Thus while we may top out at 50% of the population, this is more than twice that seen in clinical offices.

D. Study Design or Approach

Research Strategy

The proposed study is a pragmatic, cluster-randomized, parallel arm, longitudinal non-inferiority trial. Schools will be randomized to receive either simple prevention (experimental) or complex prevention (control). In YR 01 of the study, we will randomize 30 schools (15 schools receiving simple prevention and 15 receiving complex prevention). In YR 02, we will randomize another 30 schools. Treatments and data collection will be provided twice per year (one visit per semester), and all children in a given school will receive the same preventive care. Following two years of data collection, participants will be evaluated for caries arrest. Participants will be followed over a four-year period to evaluate the prevention of new caries.

We will provide an oral examination at each visit and complete a short quality-of-life instrument validated for pediatric and elementary school-age children[107-110]. The NY City Department of Health and Mental Hygiene and the Department of Education will provide sociodemographic data and academic performance data of study participants.

Hypotheses

<u>The primary hypothesis is</u>: Simple prevention is non-inferior to complex prevention with respect to caries arrest, longitudinal caries prevention, and oral health-related quality of life.

<u>The secondary hypotheses are:</u> Children receiving either simple prevention or complex prevention will have reduced school absence and higher academic performance when compared to matched, non-participating children

Evidence of Effectiveness

Table 1 provides data from systematic reviews of clinical efficacy. To be explicit, “simple” prevention consists of silver diamine fluoride + fluoride varnish (Items 3 and 6, Table 1). “Complex” prevention consists of traditional sealants + therapeutic sealants + fluoride varnish (Items 3, 4, and 5, Table 1). Sealants and silver diamine fluoride both kill the causal bacteria in carious lesions, pits and fissures (albeit by different mechanisms). Both have similar efficacy. Fluoride varnish, in contrast, converts smooth surface hydroxyapatite to fluorapatite, which is less acid soluble, and thus less prone to cavitation. Both regimens, theoretically, should have an efficacy greater than 100%. We do not expect this, but do posit that they will have similar effectiveness for caries arrest and prevention. The effects on school performance and quality of life are currently unknown.

PRECIS Assessment

We assessed the final design using the PRECIS-2 instrument[111]. Design team members (N=8) individually assessed the study protocol based on the parameters identified in the PRECIS-2 instrument. PRECIS indicates that this is a largely pragmatic trial (**Figure 5**). Deviations from the pragmatic designation arose from questions on: 1) organization – the expertise needed to efficient deliver care; 2) delivery – the requirement to deliver care twice yearly; 3) adherence – explicit site monitoring; 4) follow up-ability to access children leaving participating schools.

Figure 5 - PRECIS Assessment

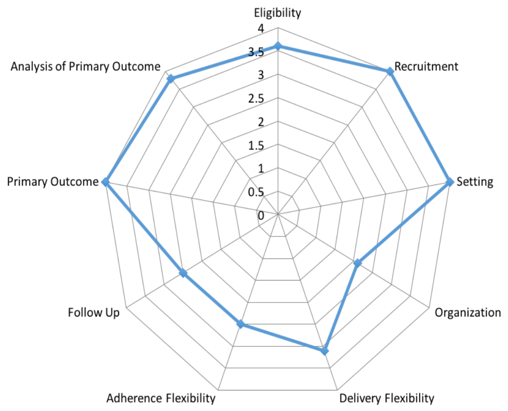

Specific Aims for Primary Hypothesis

a) Determine the non-inferiority of simple to complex prevention for caries arrest.

b) Longitudinally compare the effectiveness of both simple and complex in the prevention of new caries (incidence) and quality of life.

c) Longitudinally compare children receiving simple or complex prevention to untreated children in academic performance and school attendance.

Technical Merit

1. <u>Adherence to Methodology Standards.</u> For the enumerated items below we explicitly articulate the specific PCORI methodology standards. In this section we identify standards not addressed in the PCORI standards.

As discussed previously, national surveys, together with our literature review and key informant survey [14-16,54], indicated that there are recommendations for school-based caries prevention programs but there are no standards based on clinical outcome measures of effectiveness. In contrast, our work indicates that complex prevention reduces caries prevalence and risk[72,73]. Therefore, complex prevention is our standard of care against which we will compare simple prevention[54,112].

For clinical assessment, we will train and standardize dental hygienists and nurses in the detection of dental caries and sequelae using validated criteria implemented by the Centers for Disease Control and Prevention (CDC) in the National Health and Nutrition Examination Surveys (NHANES). Training and validation of non-dentist health care professionals and a simplified protocol was developed and documented by our Co-I, Dr. Beltran, while at the CDC and field tested in the examination of special athletes[113,114]. Training and standardization of these examiners implement the World Health Organization guidelines[115,116].

Quality of life for study participants will be assessed using the Child Oral Health Profile, short form (COHIP-SF) [107]. The COHIP measures oral health, functional well-being, social-emotional well-being, school-environment, and self-image. The Co-PI, Dr. Ruff, previously led a validation study of a preschool version of the COHIP (COHIP-PS) [108-110].

School performance (school absence/standardized test scores) are provided by the NYC Department of Education.

2. Formal Study Protocol Plan (RQ-2). As indicated in section E. Engagement Plan, and support letters, the current protocol evolved organically through iterative discussions and surveys with parents and stakeholders, beginning in 2013. We based the design on several key points that emerged from this work: 1) focus on prevention; 2) provide care in schools; 3) provide “free” care; 4) reduce cavities; and 5) improve academic performance and quality of life. In addition, a school nurse suggested that nurses should be the ones who provide simple prevention since they can already provide fluoride varnish [117]. We thus include nurses in the clinical team and collaborate with NYU Nursing on this application.

School selection and study participants. As indicated under Study Population, we worked with the NY City DOE to identify principals of elementary schools serving Hispanic/Latino children from low-income families that would like to provide caries prevention for their children. A list of participating schools is included with this application. All students with informed consent from participating schools are eligible to participate.

Care Scheduling and Location. Care is scheduled with each principal. The location of care delivery within each school is flexible – e.g., an empty classroom, on a stage, in a lunchroom or hallway, or in a nurse’s office. The school will designate the space. Care typically takes 5 minutes per child for simple and 20 minutes for complex prevention. No injections or drilling are needed.

Data Collection, Calibration, and Care. As described in item 1 in Technical Merit, clinical assessments use standardized criteria for caries diagnosis articulated in the National Health and Nutrition Examination Survey III [118,119]. Before the start of each elementary school year we will calibrate for assessment and standardize for care, both dental hygienists and nurses, in the NYU Nursing simulation center[72]. These clinicians will provide evaluation and care to school-children twice per year. We record all data on electronic dental records using a protocol that we developed in our prior and current work [16,72,73].

Formal Protocol. The formal study protocol plan will follow the guidelines and formats for clinical trials established by the National Institute of Dental and Craniofacial Research (NIDCR)[120]. The guidelines and templates include: protocol templates; study-associated documents (e.g., informed consent forms with version control, a manual of procedures, meeting agendas and minutes, standard operating procedures); data safety monitoring guidelines (e.g., charter, conflict of interest, report template); an essential documents binder (e.g., checklist, responsibilities, training log, site screening, enrollment log, monitoring log, subject code list, telephone log); data management tools (e.g., data management plan, enrollment report, odd data/errata); clinical start-up protocols (e.g., guidelines, agenda, task distribution, clinical monitoring plan); quality management documents (e.g., quality management plan, report template, consent form tracker); clinical conduct guidelines (e.g., unanticipated problems, serious adverse events, protocol deviation, consent process); and study completion protocols (e.g., close-out check list).

3. School selection and study participants (PC-2). As indicated in section C. Patient Population, we worked with the NY City Department of Education to identify, and solicit, principals of elementary schools serving Hispanic/Latino children from low-income families that would like to provide caries prevention for their children. A list of the

participating schools and principals for YR01 is included. Because principals move year-to-year, we are only providing schools for YR 01. We will again solicit principals for YR 02.

We will solicit children in the participating schools as also described in the section *C. Patient Population*. That is, our research team, will work with school parent coordinators to distribute and retrieve informed consent at the beginning of the school year, along with other informed consent.

For informed consent, the NY City DOE provides us with an electronic roster for each school. We use this roster to preprint the informed consent with a student's school, grade, teacher, name, contact information, sex, date of birth, unique identified, and bar code. Our team works with the school's parent coordinator to distribute these to each class room, and then collect the forms. The collected informed consents are scanned to the Data Coordinating Center, and the information uploaded to our electronic dental records on iPads. All patient information from that point forward is carried electronically. All examinations and treatments are securely uploaded to the Data Coordinating Center at the end of each day for cleaning and storage.

4. Interventions and Comparators (RQ-5). There are a handful of caries preventive measures for which there is evidence of efficacy (Table 1). As described above, and more fully in section *E. Engagement Plan*, we arrived at the combination prevention through iterative conversations with parents and stakeholder partners. Both arms use two approaches to caries arrest and prevention. Simple uses silver diamine fluoride to arrest caries, and fluoride varnish to prevent new decay. Complex uses sealants to arrest decay and fluoride varnish to prevent new decay. Thus, both arms have the same two aims but differ in how they arrest current decay.

5. Outcomes of Interest (RQ-6). As indicated above, for Hispanic/Latino school children from low-income families living, parents prefer: 1) Free school-based care to avoid dental office visits; 2) Prevention to arrest caries and prevent cavities; and 3) improved school performance (reduced school absence and improved test scores). These preferences mirror those of other stakeholders, including parent coordinators, and teachers (Table 2). They also mirror the interests of school administrators, and the NY City Department of Education to address the significant needs of this population[5,10,21], and the impact these health needs have on school performance[22-25].

6. Patient Reported Outcomes (PC-3). The key significant primary patient reported outcome is quality of life, specifically toothaches. We will employ the validated Child Oral Health Profile, short form (COHIP-SF) [107] to initiate conversations with each child and assess their oral health related quality of life.

7. Data Analysis (IR-3). Our data analysis plans address both primary (caries arrest, caries prevention) and secondary (academic, quality of life) outcomes, focusing on the non-inferiority of simple prevention in the treatment of existing caries (caries arrest) and in the comparative effectiveness in the prevention of new caries (caries incidence/prevalence).

For the non-inferiority of simple prevention in caries arrest, we will first determine the per-patient proportion of carious lesions treated with simple versus complex prevention that stayed arrested over a two-year period from study start. Any deciduous teeth with treated carious lesions that are lost due to exfoliation will be considered as arrested throughout the lifetime of the tooth, with arrested caries status being carried over throughout. Thus, tooth-level indicators are able to be present for both primary and permanent dentitions at the same time. With this approach, each carious tooth treated with either simple or comprehensive prevention is a trial with outcomes either of caries arrest (1) or failure to arrest (0). The percentage of arrested caries (at the child level) will thus be modeled using multilevel binomial regression with a logit link ($Y_j \sim Bin(\pi_j)$, $E(Y_j) = \pi_j$; where π_j is the probability of success).

Our noninferiority margin, δ, is set at 10%. While there is no gold standard criterion for the selection of this margin, we have set the margin based on conversations with clinicians to determine what is considered as clinically unimportant. Our null hypothesis is thus that the experimental treatment (simple prevention) is inferior to the standard treatment (complex prevention) by at least δ: $\pi_{simple} - \pi_{complex} \geq \delta$. Our alternative hypothesis is that $\pi_{simple} - \pi_{complex} < \delta$. As previously discussed, noninferiority designs are valuable when novel treatments are developed that are easier to use, are less costly, or can increase access to care. Noninferiority designs, by definition, require a consistently effective treatment to serve as the active control [121].

Based on results from the multilevel binomial models, we will use differences in effect sizes as estimated by confidence intervals to determine clinical non-inferiority of the two prevention methods [121]. We will estimate the confidence interval for the difference between the two interventions, with the width of this interval signifying the extent

of noninferiority. If the difference between the two interventions lies to the right of δ, then noninferiority will be concluded. This is the method preferred by CONSORT guidelines, however we will also provide p-values in keeping with other recommendations [121]. Following ICH E10 and CONSORT guidelines, the noninferiority component of this trial will be conducted using intent to treat analysis, including all participants as randomized regardless of their actual treatment.

For the prevention of new caries, we will use generalized estimating equations (GEE) with a logit link (caries prevalence) and a negative binomial link (caries incidence), assuming an exchangeable correlation matrix, to evaluate longitudinal effects of comprehensive care on untreated decay. We will identify the number of teeth at risk for each child during each follow-up interval and determine the number of those teeth in which new caries is observed at the examination that ends that interval. Primary teeth lost in each interval and new permanent teeth will not contribute to data for that interval. Data from baseline visits will be omitted from analyses, and will be used as an indicator of any untreated decay at baseline. We will include any relevant confounders identified *a priori* including: gender, previous (or concurrent) dental treatment (identified as new or existing treated dentition at examination), age at examination, race/ethnicity, and any school-level indicators (IR-1, CI-4). As these data have a multilevel structure, we will additionally assess caries incidence and prevalence using multilevel mixed effects Poisson and logistic modeling (ML-MEM). We will examine the effects of comprehensive prevention at multiple levels (tooth, child, grade, and school). In this analysis, we will be able to explore the variation in clinical outcomes across child and school levels. For all GEE and multilevel models, we will conduct analysis for outcomes (caries prevalence and incidence) measured by all teeth, all adult teeth only, and all deciduous teeth only. Multilevel analysis will be conducted using Stata v14.

To explore non-linear trends in untreated decay between simple and complex prevention, we will use generalized additive models (GAMs) with non-parametric smoothers. Our use of GAMs will extend ML-MEM models by linking the known known proportion p_{it} = E(y_{it} = 1 | x_{ijt}, z_{it}) to a nonlinear nonparametric predictor using the link function $n_{it} = g(u_{it}) = \ln(u_{it}/1 - u_{it}) = \sum_{j=1}^{p} s_j(x_{jit}) + z_{it}^T u_i$, where s_j are smooth nonparametric functions and u_i are random effects assumed to be iid ~$N(0, D(\psi))$ [122]. Heterogeneity and correlation among subjects will be accounted for through the inclusion of random effects. In estimating the GAMs, we will define a linear mixed model with a variance component that controls for the amount of smoothing for each additive component. We will first fit a standard mixed effects multilevel model predicting (a) the prevalence of untreated decay and (b) dmft, including all relevant confounders, and produce partial residual smoothing plots to explore any degree of nonlinearity in prevention over time. We will then introduce nonparametric smoothing terms for prevention to more adequately reflect this nonlinearity and compare models using the Akaike Information Criterion (AIC). Additional comparisons of model fit will be conducted using generalized cross validation (GCV) and unbiased risk estimator (UBRE) scores. Finally, we will allow each case to have its own smoothed trend by introducing an interaction between visit and participant. We will model case as a random effect without an autoregressive structure, imposing instead a compound symmetry correlation structure. Thus we assume that all observations from the same participant are equally correlated with each other [123,124]. Following model fit, we will produce lattice plots of autocorrelations for residuals at any lag time of preventive visits. Analysis will use the mgcv and gamm4 packages in R.

Longitudinal effects of simple and complex prevention on academic outcomes, compared to untreated children, will be analyzed using propensity score-matching and multilevel modeling. First, we will estimate propensity scores for each participant at baseline, establishing the probability of treatment assignment conditional on observed covariates (e.g., prior academic performance). Propensity scores will be used to match treatment students to students not receiving treatment, considering multiple forms of matching such as nearest neighbor and caliper. Potential comparator students will be drawn from “peer-schools”, schools identified by the NYC Department of Education as similar to treated schools based on socio-economic, academic performance, and teacher-quality indicators. This data is anonymized and can be used without consent as a secondary data sources. The Office of School Health, a collaboration between the NYC Department of Health and the NYC Department of Education, will provide student-level data for concurrent study years. Treated students and matched comparators will then be analyzed using multilevel mixed effects linear regression (for academic achievement) and Poisson regression (for school absences). If a different mechanism drives initial versus continued absences, we will analyze school absences using zero-inflated negative binomial multilevel modeling.

Comparative effects of simple versus complex prevention on child quality of life will be analyzed using multilevel mixed effects regression. Baseline quality of life will be included as a covariate and models will include predictors for

time, treatment, and the treatment-time interaction. A priori confounders, including sociodemographic variables at the child and school level, will be included.

Clinical and quality of life data collected for analysis will be housed at the Boston University Data Coordinating Center (DCC), whom we have previously worked with in multistate studies of school-based caries prevention programs. The DCC has been a data management resource center since 1984 and during this time has participated in hundreds of research projects, including multi-center and international clinical trials. With extensive experience in data management and statistical analysis, their responsibilities routinely include: design and creation of data collection protocols including case report forms and data dictionaries; subject and data tracking systems; training site personnel in data management and quality control procedures; site monitoring/auditing procedures; creation of coding manuals and Manuals of Operation; project web site design and management; design and implementation of a wide variety of data entry systems including web-based and scanning technology, with built-in range and verification capabilities; statistical analyses; and study closeout. They will ensure the highest data quality for the proposed study. Designing and implementing the systems for collecting, entering, reviewing, cleaning, tracking and reporting will be accomplished during Year 1; maintenance, revision, troubleshooting and data cleaning will occur during Years 1-4; preliminary analyses will occur during Years 2-5; creation of final, clean data sets and statistical analysis will occur during Year 5. Further information can be found in **Consortium & Contractual Arrangements**.

Educational data is available via cleaned historical records managed by the Research & Policy Support Group (RPSG) at the NYC Department of Education and the Office of School Health at the NYC Department of Health. The co-PI of this study, Dr. Ruff, was previously the Director of Research & Evaluation at the NYCDOE and the Director of the Research & Evaluation Unit at the NYCDOH, with a concurrent appointment as a senior research scientist at the RPSG. Student level data (including demographics, academic performance, and school attendance data) are available each year for every student and trackable using standard identification numbers.

Secondary data analysis: Following completion of the CariedAway trial, we will perform additional analyses through a supplementary project from 1 October 2023 to 30 September 2024. These analyses include:

Estimate the incidence rate of caries over time in the presence of interval-censored data. For each visit we will calculate the time elapsed from the prior visit, document the health status of every tooth, and compare it to the status at the previous visit. This will generate an initial aggregate of person-time and disease incidence, by visit, for each treatment group. To properly estimate incidence rates in the CariedAway treatments (SDF, sealants, and atraumatic restorations), we will apply the two-stage multiple imputation method of Vandormael et al (2020) [125] ("g-imputation"). Stage one will utilize semi-parametric G-transformation to estimate the cumulative baseline hazard function. Stage two will derive the cumulative distribution function for each participant, imputing infection time conditional on covariates that are selected to improve accuracy of rate estimates. The Vandormael g-imputation method has been shown via simulation to out-perform deterministic imputation methods (e.g., mean imputation), but has not yet been applied to oral health data. In our application to CariedAway, we will utilize time-independent (e.g., sex, race/ethnicity, treatment assignment) and time-dependent (severity of baseline decay, age at time of examination, etc.) variables as our conditional covariates in estimating annual incidence rates. Incidence rates will then be calculated using the standard methods: the total number of new disease in treatment groups will be calculated and divided by the total observed person-time. These individual rates will be divided to produce rate ratios. We will consider alternative imputation methods that preceded g-imputation in our analyses, including the Cox proportional hazards/non-parametric estimation method of Hsu et al (2007) [126] and the multiple imputation approach of Pan (2000) [127], as well as comparing results from g-imputation to standard mean imputation as a sensitivity analysis.

Identify the overall clinical efficacy of SDF when used in a pragmatic setting. This encapsulates a number of questions to refine the applicability of SDF in school-based care. **RQ1**: *What frequency of SDF application is necessary to successfully arrest caries?* We previously observed that a subset of children receiving silver diamine fluoride for dental caries fail to respond to treatment (nonresponse), developing new carious lesions after initial application. We subsequently demonstrated that the differential abundance of select microbiota and specific pathway functioning in individuals that present with recurrent decay after SDF treatment may contribute to a potential failure of silver diamine, and observed the highest abundance of phosphotransferase system and lowest abundance of lipopolysaccharide synthesis in non-responders [128]. We further developed a model using artificial neural networks to predict treatment nonresponse [129]. However, while this model can be used for early identification of potential nonresponse, its complexity

and data requirements imply that practical, chairside solutions may be more suitable. Application frequency protocols for SDF vary extensively among dentists, from once per week for 3 consecutive weeks (once per year), to once at the diagnostic visit, then at 1 and/or 3 month follow ups, then at semi-annual recall visits (6, 12, 18, 24 months). Caries arrest has been found to increase dramatically after re-application, suggesting that multiple applications are more effective than single applications (as well as for prevention). In this aim, we will use the Caried*Away* dataset to identify those at baseline receiving SDF for dental caries, and subsequently assess in subsequent observations what percentage fail to arrest or prevent caries (non-response). We will then determine how many additional treatments with SDF were required to recapture previous SDF non-responders. Analysis will use mixed effects multilevel models. **RQ2**: *Does indirect application of SDF on caries result in arrest?* SDF has been determined to possess the combined effects of silver and fluoride, namely, antibacterial and remineralization effects [130]. Silver ions in SDF are thought to prevent bacterial aggregation through reaction with bacterial cellular surface proteins [131], harden soft carious lesions through reaction with phosphate or chloride ions resulting in the formation of silver salts (e.g., silver chloride), and increase the alkalinity of the environment through the formation of ammonium compounds hypothesized to have an acid-buffering effect [132]. Whereas the direct effects of SDF is a topic of considerable review [133-135], the indirect effects of its application on salivary composition, oral biofilm, and caries activity have not been meaningfully examined. In this aim, we will use the CariedAway data to assess the indirect effects of SDF on caries activity, specifically arrest. First, we will identify all subjects that presented with dental caries on both posterior and anterior teeth. Since, in CariedAway, silver diamine fluoride was applied only on posterior teeth, we will assess all subsequent observational periods and determine if anterior decay was arrested following indirect application of SDF. The rates of SDF indirect arrest will be analyzed using multilevel mixed effects regression with subjects being groups by the severity of baseline decay on posterior teeth as an indicator for the magnitude of SDF applied. **RQ3:** *What are the transition probabilities for children receiving SDF in a school-based program?* Transition probabilities can be useful for planning and implementation strategies of school-based programs, can be used to predict caries in long-term observational cycles [136], and support cost-effectiveness research [137]. In this sub-aim, we will apply nonhomogeneous Markov models to analyze longitudinal caries transitions in children receiving silver diamine fluoride using the CariedAway data [138]. We will analyze both the transition from caries free to carious and from those initial carious at baseline, arrested from treatment, and subsequent recurrence. Models will be stratified by the total treatments received in each group.

C<u>ompare the time required for treatment between silver diamine fluoride and glass ionomer sealants, and identify how time varies by provider type and severity of need</u>. Estimating the true population-level impact of school-based caries prevention using SDF or sealants requires knowledge of the time required to treat children, and also informs how much time is required outside of the classroom for care. These estimates, when combined with the clinical efficacy in Objective #1, can support identifying the population health impact of caries prevention. ***Statistical Assessment**:* as part of our electronic oral health record system (Figure 3), we are able to automatically generate time stamps for every stage of the examination and treatment procedure. For example, the steps of the CariedAway data collection procedure consists of (1) a sign-in page, (2) consent and demographic information page, (3) screening/examination, (4) treatment, and (5) referrals and proof of treatment. Every entered data point in the system has a corresponding timestamp. Using these time-stamps, we will calculate for each person and each visit how long individual phases the data collection process took (e.g., assenting, clinical examination, treatment, etc.). The total time for each observation and person will be stratified by treatment group (SDF versus sealants/ART), total severity of baseline decay (e.g., no decay, 1 decayed tooth, multiple decayed teeth), age of the child, and mix of dentition (proportion of deciduous vs permanent). For subjects treated with SDF we will also determine treatment time by who is providing the care, as CariedAway included both nurses and hygienists giving care. Once the total time is calculated, differences between groups will be assessed using multivariate analysis of variance. Should the standard MANOVA assumptions not hold, we will utilize permutation tests (minimum permutation of 10,000) and Aligned Ranks Transformation ANOVA [139,140]. Post-hoc comparisons will be adjusted using the method of Benjamini & Hochberg [141] to control for the false discovery rate. Statistical significance will be set at 0.05. For longitudinal analyses by subject visit, data will be analyzed using mixed-effects multilevel regression models (ME-MLM).

Power analysis: The study is powered to the primary outcomes of caries arrest and caries prevention. We expect to enroll approximately 10,573 students across a maximum of 60 schools over the duration of the study (N=176 per cluster).

We note that the primary enrollment target of 10,573 is agnostic to the maximum school enrollment as within-school subject recruitment is expected to vary from school to school. Full study population may be enrolled from less than the maximum school recruitment target. From our pilot studies of school-based caries prevention, baseline caries prevalence is approximately 40%. Our power estimates assume an equal proportion of success, π, of 20% caries arrest. With a given non-inferiority margin (δ) of 10%, a total sample size per group of 198 (N_{tot} = 396) is required for an alpha of 5% and a power of 80% [142] . However, as we use a cluster randomized design, this sample size is inflated to account for clustering (assuming a very conservative intraclass correlation coefficient of .10) by a design effect of 18.5 to a total required sample of 7,322. We expect to encounter a 20% attrition rate in our total enrollment due to patient loss to follow-up and maturation of children (graduation from school). However, we further expect that in each year of the study this attrition will be partially offset by newly enrolled children who enter the school.

For caries prevention, both interim therapeutic restorations and silver diamine fluoride have been shown to be 80% efficacious in individual clinical trials [5,6]. Power for the repeated measures design was estimated using the method of Diggle et al (2002) for generalized estimating equations [143]. For power estimates, we control for the baseline prevalence of untreated decay. We assume a conservative average number of visits per child of 6, with a power of .80 and an alpha of 5%. We further assume a repeated measures correlation of 0.5. For a given minimally detectable effect size (standardized effect size difference) of .25, sample size of 146 per arm is required, or a clustering adjusted sample size of 3,562 per arm. Thus, our study is powered for these conservative assumptions for caries prevention even assuming moderate subject attrition. Further, we note that in the presence of the nonlinear link function, ME-GLM is more powerful than GEE, thus our power estimates are conservative and the anticipated sample size is sufficient for analysis.

For secondary outcomes, for the 2013 school year, the citywide average absenteeism rate was 14.24%. For power calculations, we use a simple two-group cluster randomized comparison of means (standardized test performance, quality of life) or proportions (school absences). Power estimates adjust for the design effect due to cluster randomization. Similar to estimates for untreated decay, we assume an intraclass correlation of 10%, power of .80, and an alpha of .05. For a simple clustered two-group comparison (simple versus standard of care or complex versus standard of care), a sample size of 14,100 is expected to detect a 9% decrease in absenteeism (to 5%). Additionally, the 2016 average citywide performances for reading and math examinations (grades 3-5) were 298 (SD=17) and 299 (SD=21), respectively. The New York City Department of Education regards a 3% change as significant. For an ICC of 10%, power of .8, and an alpha of .05, the sample size is powered for a detectable difference of 6.44 (reading) and 7.95 (math). Quality of life will be measured using the COHIP-SF. In a validation study of the COHIP-SF, a pediatric sample of children (aged 7-17) had average COHIP-SF scores of 56.2 (SD=9.3). For a simple cluster randomized, two-group mean comparison, with an alpha of .05, power of .8, and ICC of .10, the study is powered to detect a difference of 2.2 on the COHIP-SF scale. Notably, for all secondary outcomes, we will have longitudinal data and thus greater power to detect differences. It is possible that the detectable effect size or total power of the study is reduced for secondary outcomes due to the anticipated enrollment of 10,573 participants.

Power for secondary data analysis: The CariedAway dataset consists of over 10000 individual records across more than 7000 subjects. This exceptionally large sample size affords a high degree of power for the proposed aims. Multilevel power calculations for methods proposed in Objectives 1 and 2 follow the method of Diggle et al. (2002) for generalized estimating equations. Estimates assume a statistical power of 0.80 and a two-sided type I error rate of 5%. A repeated measures correlation of 0.5 and a per-visit attrition rate of 20% were also assumed. For a given minimally detectable effect size (standardized effect size difference) of 0.25, a cluster-adjusted (ICC = 0.10) sample size of 12,874 records is required. In actuality, the clustering effect previously determined in CariedAway was negligible (ICC = .000314), as a result, the study is powered for these conservative assumptions for caries prevention. For Objective 3, power for a two-group independent mean time comparison with alpha of 0.5, power of 0.95, and a small effect size (d=0.2) requires a sample size of 1084, and thus this aim is powered for multiple possible analyses and interaction effects.

8. Validated Instruments (IR-4). The clinical measures to be used are those used by NIDCR [119] as modified for the National Health and Nutrition Examination Survey [118]. These measures are congruent with currently used clinical assessments by dentists nationally and globally. They include multiple assessments of primary and permanent teeth and surfaces as: missing, sound, decayed, or filled. We will also assess presence/absence of pain/swelling, and pulpal involvement. School attendance and standardized test scores are collected and reported annually by the NYC DOE. Standardized tests used by the NYC DOE are validated instruments. Quality of life for study participants will be assessed

using the Child Oral Health Profile, short form (COHIP-SF) [107]. The COHIP measures oral health, functional well-being, social-emotional well-being, school-environment, and self-image. The Co-PI, Dr. Ruff, previously published studies of child quality of life in children with cleft palate and recently validated the preschool version of the COHIP [108-110].

9. Data Source Adequacy (IR-1). Clinical data will be derived from clinical examination by standardized examiners. Educational data will come from records maintained at the Office of School Health, housed at the NYC Department of Health and the NYC Department of Education. These records contain individual-level data for a complete census of K-12 students attending NYC public schools from the 1995-96 through 2016-17 academic years (a universe of roughly 1,000,000 observations per year) and for this project it will be current through 2022-2023. Every student record contains detailed demographic, program and academic information including birthplace, race, gender, language ability, attendance rates, participation in school breakfast and lunch, admit/discharge dates and codes, participation in special education and language programs, and standardized test scores in English Language Arts and Mathematics for students in grades 3-8. We will have data on students in both the experimental and the comparator group. These student-level data are combined with publicly available data from the *Annual School Reports, School Based Expenditure Reports* and *State Report Cards*, prepared annually by the NYC DOE and the New York State Education Department. There are no specialized resources or capabilities required to analyze the above data.

10. Data linkage plans (IR-2). Each student will be identified by his/her NYU Office of Student Information Services (OSIS) numbers, which are the unique identification numbers used by the NYC DOE. Each student is given an OSIS number when he/she first registers in the school system. This number is used throughout their enrollment in NYC public schools and they are re-assigned this number should they withdraw and re-enroll. We will send the NYCDOE a list of OSIS numbers for students for whom we have parental consent for participation. The NYCDOE will then scramble the OSIS numbers to create the "NYUID" number to be matched back to the database housed at the Office of School Health.

11. Sensitivity Analyses (IR-5). There are several sources of heterogeneity in the sample that might influence conclusions. Thus, we will evaluate whether results are robust by differentiating analysis according to: different levels of disease severity at baseline; different numbers of teeth at risk; data from primary vs. secondary dentition; age at entry; different times of entry into the program (consistency among recruitment waves); and finally, variable durations of time in the program. Sex and ethnicity are not considered here, as they are already evaluated as factors in planned subgroup analyses. Additionally, results will be compared across varying models, such as negative binomial versus Poisson.

12. Missing Data. *(a) Methods to prevent and monitor missing data (MD-1*): we will obtain complete data on study participant demographics from the NYC DOH and the NYC DOE. These data include birth date, race, gender, and socioeconomic status in addition to academic record data. Clinical data will be recorded at baseline and follow-up visits using electronic dental records. We will link to student demographic information using unique identifiers to ensure the highest data retention and accuracy possible.

Our electronic data collection program (for dental indicators) is designed to alert the user of missing or out-of-range assessments. Therefore, we expect the major portion of missing data to be due to the transient nature of the patient population. Student participation in the program will be monitored over the five-year study period and we will attempt to locate students who leave participating schools but stay in the NYC school district to assess educational outcomes. We will not, however, be able to do clinical assessments on children who move to a non-participating school. We will provide dental care if they move to a participating school, and the data will be analyzed based on their starting school.

Clinical data will be reviewed following each semester for data accuracy, and issues of missing data will be investigated and logged. Any issues of data "missingness" will be compared to established data collection protocols, which will be revised as appropriate to improve collection procedures. To reduce the potential for systematic missing data we are collaborating with NYC DOE in developing our research design and data analysis plan.

(b) Statistical Methods to Handle Missing Data (MD-2): we will examine the reported reasons for patients lost to follow-up and summarize the patterns of missing data according to statistical assumptions of missing completely at random (MCAR) and missing at random (MAR). Under the assumption of MCAR, the proposed GEE method is valid for inference. When the missing mechanism is MAR, the likelihood-based approach of generalized mixed-effects models will be employed to accommodate the missing data. In addition, we will utilize multiple imputation (MI) methods to represent expected variability in imputed values due to missingness. Given that the validity of results rests upon specification of the proper missing data mechanism, we will compare imputed results to results using original data to

identify any discrepancies in estimates. When missing data are present in general, we will conduct sensitivity analyses with multiple statistical models and to examine the stability of our results to various model assumptions.

(c) Plans to use Validated Methods to Deal with Missing Data That Properly Account for Statistical Uncertainty Due to Missingness (MD-3). As addressed in the previous paragraph (MD-2), we propose to use multiple imputation procedures where viable. We will also explore the sensitivity of inferences based on our assumptions by comparing results from MI-complete and maximum likelihood (expectation-maximization) datasets with that of non-imputed analysis. These methods are preferable to single-imputation methods such as regression imputation or last observation forward. A strength of our data analysis plan is that for the longitudinal analysis, our proposed models allow for missing data on time-varying covariates and thus do not require complete-case data for analysis. This maximizes our sample size and includes any information available for all participants in the study.

(d) Plans to Record and Report Dropout and Missing Data (MD-4). Attrition rates and identified reasons for attrition will be reported in statistical analysis sections of any presentations or publications when discussing sample size. We will identify any participants who drop out of the study with the informed consent log, updated each year in each school. Historically 5% of children may appear to be lost to follow-up due to school absence during the scheduled examination/ treatment period. These children usually are identified at the next semester's visit. For students that are still enrolled the NYC school district, but transferred to a different school, we will note this change. Academic and demographic data will still be available for students in the NYC school district.

(e) Plans to Examine Sensitivity of Inferences to Missing Data Methods (MD-5). We will describe missing data and compare differences in baseline characteristics of patients with and without missing data to identify any differential attrition that may bias results. We note that any assumption made of NMAR is unverifiable using observed data. Without external information regarding the underlying cause of missingness, the observed data provide no information for determining the validity of assumptions of NMAR data. Therefore, as mentioned in MD-2 and MD-3, we will explore variation in results under different missingness assumptions and using different methods of modeling and imputing missing values, particularly Multiple Imputation and MLE-EM methods. We will address the implications of these results in any formal conclusions or discussions that arise.

13. <u>Heterogeneity of Treatment Effects (HTE).</u> *(a) Goals of any Planned the Analysis (HT-1).* Our primary hypothesis addresses both average treatment effects (ATE) and average effect of treatment on the treated (ATT). Our preliminary results suggest that there is no differential response to treatment. However, secondary analyses will consider any heterogeneity of effects across groups. In particular, we are interested in the differential effects of comprehensive prevention when stratified by: initial oral health status; race/ethnicity; and pre-intervention school attendance and academic performance.

(b) Analysis Plan to Test Hypotheses (HT-2). We will identify groups as "no decay" or "any decay", as well as the number of teeth presenting with decay at baseline. In this latter group, we will include students with one, two, three, or four or more teeth with caries at baseline. We will stratify by decay at baseline and conduct longitudinal analyses for each stratum. We expect effects to be greater in patients presenting with more decay. **Figure 3** displays summary results using these methods. We further hypothesize that treatment effects will not vary with race/ethnicity. This analysis addresses the overarching hypothesis that caries is driven primarily by the biology of the infection and by socioeconomic status, and not race/ethnicity. We will incorporate relevant potentially important subgroup variables to address this question. Again, **Figure 3** displays summary results incorporating these methods.

We will conduct two primary analytic procedures. First, we will test the hypothesis that students with greater baseline academic achievement derive less academic benefit due to comprehensive caries prevention. We will stratify students based on prior academic performance and conduct individual stratum analyses. Second, we will revisit our earlier propensity score matching to identify any heterogeneity in treatment that may be due to probability of treatment assignment, conditional on listed covariates. We will analyze treatment effects within propensity score strata to explore whether there are significant differences in effects between included and excluded participants (e.g., those with high scores and those with low scores). Should we identify any heterogeneity in effects by propensity scores, further investigation will be conducted, such as subgroup analyses. To formally test and quantify the heterogeneity of treatment effects across different subgroups, we will use the Q test and the I^2 test.

(c) Basis for HTE Claims (HT-3). Any claims for heterogeneity of treatment effect will be based on appropriate contrasts: we will present stratum-specific treatment effects to use in comparing across proposed groups.

(d) Plan to Report Pre-Specified and Post-Hoc Analyses (HT-4). In any report or publication that incorporates results from HTE analyses, we will include the pre-specified group comparisons as well as note any new groups or comparisons that arise during the study. We will specify categorical predictors, cut-offs for stratification of performance and propensity scores, and clinical outcomes. For multiple comparisons, we will report on procedures used for p-value adjustment and false discovery rates.

14. Reporting Plan and Assessment of Internal and External Validity (IR-6). To ensure maximization of internal and external validity, the design team adhered to reporting guidelines identified by EQUATOR Network website [144]. These include the: SPIRIT 2013 statement articulating protocol items for cluster randomized controlled trials [145] that are extensions of the CONSORT 2010 statement for randomized controlled trials [146,147]; SAMPL statement for reporting statistical analysis and reporting [148]; and TIDieR statement for reporting interventions [149].

To ensure adequate representation of the study's internal and external validity, all of our reports will include information on design, randomization, data collection, and statistical analysis that meet the reporting guideline standards identified on the EQUATOR Network.

Not Applicable: Non-randomized study; PCOR Data Registry; PCOR Data Network; Adaptive/Bayesian Designs; Diagnostic Tests.

E. Engagement Plan

Criterion 6. Patient and Stakeholder Engagement

Planning for this study began in 2013 when the NY City Department of Education (DOE) asked the PI to implement a NY City-wide caries prevention program. Regular meetings with and surveys of parents and stakeholders, evolved our co-thinking and learning about the neediest population (Hispanic/Latino), location (Bronx), subjects (children from low income families), preferences (provision of free school-based prevention and treatment of cavities (without drilling and filling), and outcomes that measure cavities, and quality of life (e.g.: toothaches), school performance, and identify funding for a two-school pilot to determine feasibility.

The pilot, in turn, led us to: modify, simplify, and personalize the informed consent ; include nurses on the clinical team, compare simple and complex prevention; identify and include community parents and stakeholders interested in participating in the program; solidify NY City stakeholders for city-wide dissemination; and identify stakeholders for national dissemination.

1. Planning

In 2013 Dr. Marcelo De Stefano, NY City Department of Education, Director of School-Based Health Centers, Dental Clinics and Health Insurance, requested that our department initiate a NY City-wide school-based caries prevention program. Based on national, state, and city data, he advocated for a program to begin in the Bronx and focus on Hispanic/Latino children from low income families. Dr. De Stefano introduced us to leadership at the New York City and New York State Departments of Education and Health. He also introduced us to the Children's Aid Society (100-year old city-wide organization that provides educational and health support for children in low income families), and Children's Health Fund (a Bronx located national organization providing school-health nationally). Finally, Dr. De Stefano introduced us to Bronx elementary school principals. With this as a foundation, and with facilitation from Ms. Nydia Sanchez and Phoebe Christian at Children's Health Fund, we identified patient and stakeholder partners (**Table 5**) had iterative conversations and carried out surveys with Bronx school principals, nurses, parent coordinators and parents to more specifically identify needs.

The consistent community needs included: provision of free school-based prevention and treatment of cavities (without drilling and filling), improved oral (e.g.: reduction in cavities and toothaches), and improved school performance. We shared with parents and stakeholders what we might offer to address their needs, and evolved to the two proposed prevention strategies, along with the relative benefits and risks. From these co-learnings evolved a two-school pilot program and the current proposal. The research team now includes 3 Latina mothers who are Bronx

residents and half of our Patient & Stakeholder Partners are either Hispanic/Latino or Black, to assure us that the conduct and dissemination continue to address child/parent and community needs.

2. Conduct

Dr. De Stefano, along Ms. Phoebe Christian (then at Children's Health Fund, and now the Administrative Director for Community Pediatric Programs at Montefiore Health Systems, Bronx, NY) and Ms. Nydia Santiago, school health manager at Children's Health Fund assisted us in developing and implementing the pilot program and the current proposal. A key element in this evolution was Ms. Christian's strong recommendation for school-based prevention. This paralleled patient preferences, but for different reasons. Montefiore's challenges revolve around patient scheduling for clinical care and the cost of dental van costs for drivers and maintenance. For the pilot and proposed program we generated an New York University/New York City Memorandum of Understanding, an informed consent with IRB approval, and identified funding from Colgate-Palmolive and Henry Schein. Dr. De Stefano and Ms. Christian are participating in the proposed program as part of the Clinical Engagement Team and the Patient & Stakeholder Partners.

Table 5. Patient & Stakeholder Partners and Roles

Representing	Organization	Person
Latina Parents (children)		Ms. Santiago*+
		Ms. Lopez*+
		Ms. Alizea*+
		Ms. Cruz+
		Ms. Christian+
NY City		
	Department of Education	Dr. De Stefano*+
	Department of Health	Ms. Kalra
	Principals	M. Cannon+
	School Nurses	Ms. Santiago*+
	Parent Coordinator	Ms. Lopez*+
NY City Organizations		
	Montefiore Hospital	Ms. Christian+
	Children's Aid Society	Ms. A. Cruz+
Policy		
NY City	DOE/DOH	Dr. Platt
NY State	DOH	Dr. Richardson+
Insurers		
State	Medicaid	Dr. Gostling
	Healthplex	Ms. Zelkind
National	United Concordia	Dr. Bramson
Dissemination		
Policy	Pew Trust	Dr. Maas
Nursing Education	NYU	Drs. Haber/Hartnett
Media	Univision	Dr. Niederman

* Denotes dual role; +Denotes Black/Hispanic Race/Ethnicity

The pilot program introduced us to unique aspects of the New York City schools. This included access to electronic school rosters with demographic information on each student and their unique identifier. Ms. Santiago suggested, and we created, personalized informed consent for each child with their school, grade, teacher, name, and date of birth. We created these on TeleForm so we could more easily distribute these by class, and after collection scan the information into our electronic dental records for longitudinal tracking.

Ms. Sanchez, who is a Latina mother of school age children and a registered nurse, participated in the pilot program. She recognized that nurses, and particularly school nurses, could provide simple prevention and suggested that we have nurses on our clinical team. We now propose this, and Ms. Sanchez is a Co-I and will lead our Clinical Engagement Team. One school coordinator, Ms. Nicole Lopez, who participated in the pilot shared how each school has a parent coordinator who would need to be trained if we were to disseminate this program. She is now a part of the proposed Clinical Engagement Team.

The proposed program now engages the NYU Colleges of Dentistry and Nursing, includes parents and representatives of community organizations, implements and assesses interventions and outcomes of import to the recipients of care.

3. Dissemination

Broadly, the proposed program uses standards from systematic reviews for dissemination and implementation and from the National Institutes of Health and the Centers for Disease Control and Prevention for the manual of procedures

and assessments. More specifically, as indicated at the outset of this section, and as indicated in his support letter and those of Dr. Platt and Ms. Kalra from the NY Departments of Education and Health, support New York city-wide dissemination. Data emerging from this initiative will enable us to determine if the two protocols are similar for all age children, and if they are not which might be better for which age group. The inclusion of NY State Department of Health Dental and Medicaid director, as well as a Healthplex dental insurer should assist us with broad visibility at the policy levels, and therefore greater opportunity for dissemination.

We will pursue academic manuscripts and conference presentations. But more effective dissemination and implementation will be done in collaboration with the Children's Health Fund. They participated in creating the pilot program and are interested in learning how the more successful (or both) prevention protocols can be implemented in the 13 states where they operate. Our NYU Nursing collaborators, who are Co-PI and Co-I, are also interested in updating and broadening their national caries prevention training programs. And finally, on September 1, 2016, NYU Dentistry announced a partnership with Univision Communications, the leading media company serving Hispanic America, to promote improved oral health among Hispanics[150]. The PI is the editorial director for the NYU Dental-Univision partnership.

F. Research Team and Environment

Criterion 4. Investigator(s) and environment

The leadership team (PI, Co-PIs, Co-Is) have significant, and complementary skill sets for caries prevention training, implementation, and evaluation. This includes study design, clinical implementation, and analysis. The dental PI, a Co-PI, and a Co-I, have a career focus on caries prevention and implemented and evaluated caries prevention programs locally, nationally, and globally from 3 to 25 years, with a total of 43 years of experience between them. The nursing Co-PI, and 2 Co-Is have careers focused on caries prevention , and have national caries prevention teaching and implementation experience ranging from 3 to 8 years, with a total of 16 years of experience between them.

We provide an organizational chart in the Budget Justification.

The proposed work is supported by both the deans of the NYU College of Dentistry and the College of Nursing.

Research Team

The PI, Dr. R. Niederman began implementing school-based caries prevention more than 10 years ago in Massachusetts [72], and continues to work: expanding the caries prevention evidence base [54,69,112], promoting and expanding implementation of school-based caries prevention [6,53], identifying barriers to effective caries prevention [16], and demonstrating clinical effectiveness of caries prevention [16,73]. For this work he continuously collaborated with New England Survey Systems in creating secure mobile electronic dental records (currently on iPads), and with the Boston University Data Coordinating Center for data cleaning, storage, and preliminary analysis. The current clinical design and analytic approach began two years ago with the FDA's approval of silver diamine fluoride use in the U.S. The PI's knowledge of silver diamine fluoride for simple prevention [69,112], review of multiple caries preventive measures [54], and experience with complex prevention [16,72,73] all led to this proposed comparative effectiveness study of simple and complex caries prevention.

The Co-PI, Dr. R. Ruff, is an epidemiologist and statistician with experience in the relationship between oral health and child development, including academic achievement, psychosocial functioning, and quality of life. His recent work focuses on the analysis of school-based caries prevention programs, including nonlinear trends of prevention on untreated decay and causal effectiveness. He has extensive knowledge and experience in research design, statistics, and the analysis of dental and educational data. His previous position was Director of Research and Evaluation at the NYC Department of Education and Director of the Research & Evaluation Unit at the NYC Department of Health and Mental Hygiene, where he conducted city-wide research studies on educational performance and chronic diseases. He will oversee the Analytic Team.

The Co-PI, Dr. J. Haber has extensive experience and expertise in interprofessional education. In particular, her most recent work focuses on interprofessional education and practice, with a special focus on oral-systemic health for registered nurses, nurse practitioners, and midwives. Dr. Haber is the Executive Director of two national nursing oral

health initiatives, the *Oral Health Nursing Education Practice (OHNEP)* Program funded by the DentaQuest and Washington Dental Service Foundations, and Principal Investigator on the HRSA-funded *Teaching Oral-Systemic Health (TOSH).* She is a member of the Steering Committee of the National Interprofessional Initiative on Oral Health (NIIOH), a member of the Technical Expert Panel that developed the 2015 White Paper, *Oral Health: An Essential Component of Primary Care.* She was a member of the HRSA Expert Panel that developed the 2014 *Interprofessional Oral Health Core Competencies for Primary Care Providers*. She, with Dr. Harnett and Dr. Beltran, will develop and implement the training program for silver diamine fluoride.

The Co-I, Dr. Harnett, a certified Pediatric Nurse Practitioner (CPNP), is the Program Director for NYU Nursing's oral health programs: Oral Health Nursing Education and Practice (OHNEP) and Teaching Oral Systemic Health (TOSH)**.** She facilitates the ongoing commitment of NYU Nursing and NYU Dentistry and NYU Medicine leadership to interprofessional oral-systemic health competency development and curriculum innovation. In these contexts, she developed an oral health education program for the Nurse Family Partnership that is nationally endorsed. She is expert at training Registered Nurses, Nurse Practitioners, Midwives, and Physicians to integrate interprofessional oral health competencies into their clinical practice as a standard of care, including oral assessments and topical fluoride varnish application. She, with Dr. Beltran will oversee training, calibration, and standardization of clinical assessment and caries prevention.

The Co-I, Dr. E. Beltran, is a pediatric dentist and epidemiologist, who also serves as the Executive Director of the American Board of Dental Public Health. His major research areas include dental caries, remineralization of caries lesions, pit and fissure sealants, dental fluorosis, and surveillance of oral diseases. He has trained over 200 examiners for major national surveys in U.S., Latin American and Asian countries, using either the World Health Organization or the National Institute of Dental and Craniofacial Research protocols. Between 1999 and 2014, he was the lead and then senior epidemiologist for the CDC National Health and Nutrition Examination Survey. As a Senior Director of Scientific Strategies, Information, and Emerging Issues at the American Dental Association (ADA), he had scientific oversight for the ADA Council on Scientific Affairs, the Center for Evidence-Based Dentistry and the Scientific Communications Program of the Division of Science. With Dr. Hartnett, he will oversee training, calibration, and standardization of clinical assessment and caries prevention, and collaborate with Dr. Ruff for analysis.

The Co-I, Ms. N. Santiago, is a registered nurse, and Latina mother of school age children. She is currently a Senior Site Manager at the Children's Health Fund, where she works to coordinate screening and care for asthma, hearing, vision, and dental. In this context she facilitated meetings with parents and school faculty, arranged for informed consent in the pilot schools, and actively participated in the pilot program. She will join the NYU team as a Co-I, oversee the Community Engagement Team, and Co-Chair the Patient & Stakeholder Partners.

Community Engagement Team is entirely Latina/Latinos. The individuals are: Ms. Santiago, Ms. B. Alicea, Ms. N. Lopez, and Dr. De Stefano. Ms. Alicea is trained as an engineer, but changed careers to focus on community work. As our Clinical Coordinator, she will coordinate day-to-day clinical care. Ms. Lopez is currently a parent coordinator, and will join the team as a School Coordinator. She will communicate facilitate communication with parent coordinators at each school for informed consent and follow on care. Dr. De Stefano works for the NY City Department of Education and will consult with the Engagement Team to overcome unforeseen hurdles.

Patient and Stakeholder Partners. To ensure community voices are heard during planning, conduct and dissemination of the program we assembled a group of Patient and Stakeholder Partners. The 13 members will quarterly on Skype, and in-person once yearly. Prior to these meetings, the PI will provide members with a summary report. The report will consist of recruitment, retention, and analysis.

The partners are identified above in **Table 5**. Among the partners, 5 are Latina/Latino, and 3 are Black. Among the Latinas, 4 are mothers of school-age children. Letters of support and resumes are provided with the application. The budget justification and resumes provide particulars on each individual.

Analytic Team. Dr. Ruff will lead the analytic team. He will be joined by Dr. Beltran and one of our research faculty statisticians, Dr. Janal.

Data Coordinating Center. We've worked with the Boston University Data Coordinating Center on school-based caries prevention for 10+ years. The team includes Christine Chaisson, MPH, Principal Investigator of the Subcontract

who will supervise all Data Coordinating Center personnel and activities to insure that datasets are clean, fully de-identified, and accurate. Joseph Palmisano, MPH, MS, Statistical Analyst is a proficient SAS programmer and data analyst, and is the analyst for the project. He will create de-identified dataset which will entail removing all identifiers including dates which will be replaced by year/semester indicators and number of days between visits for instances when a child has more than one visit in a calendar year. Final de-identified datasets will be created in SAS and SPSS and will be available as CSV files if requested. Leah Forman, MPH, Data Manager**,** is an experienced data manager and SAS programmer and serves as the data manager on the project. John Lu, MS, Systems Analyst**,** is Assistant Director for Web Applications at the DCC and is a proficient web and database programmer. Mr. Lu is the systems analyst on the project.

Dean's Support. A letter of support, signed jointly by the deans of the NYU College of Dentistry and the College of Nursing is included with this application.

Research Environment

New York University was founded in 1831 as "a private university in the public service." The current project proposal continues this tradition by focusing on providing preventive oral healthcare to NY City's poorest children. The research teams have access to all university facilities and resources. With an enrollment of 50,000 students and five million square feet of interior space on campus, NYU is one of the nation's largest private universities. It has 13 schools, colleges, and divisions. NYU maintains seven libraries containing over 4.5 million volumes. A computerized catalog provides access to most of the libraries' holdings. The main collection continues to grow by more than 140,000 volumes a year. NYU also has six specialized libraries that contain over 168,000 volumes, 2,000 periodicals, computer software and audiovisuals. The NYU Television Center, which also acts as a resource, manages satellite downlinks for instructional and educational programming, and provides on-campus teleconferencing. NYU provides networked PCs and Macs with Internet access and electronic mail capability to all faculty and staff. A computer technical help desk and full-time network administrators are available for assistance and trouble-shooting through Information Technology Services. The University also has six computer labs and special resources for media production, arts technology, and science and humanities computing. The information technology department provides particularly strong resources for social science and statistics programs that include lectures, workshops, and expert consultations. The NYU wireless network continues to expand into new areas of the campus.

The NYU College of Dentistry and the NYU Rory Meyers College of Nursing are two of the leading dental and nursing programs in the country. NYU Dental is ranked #10 among dental schools for NIH funding, and NYU Nursing is ranked #8 among nursing schools for NIH funding. Both schools have robust outreach programs, both in New York City and globally, serving vulnerable populations across the lifespan.

NYU Dental currently provides care in 10 elementary schools and 20 Head Start programs. NYU Nursing provides primary care for 8 high schools in Brooklyn, including two international high schools serving a recent immigrant student population. NYU Dental and Nursing partner at the international high school and a foster care agency in Queens.

NYU Dentistry and Nursing teams reside in the same new building on First Avenue on the NYU Health Science Corridor, and share floors. The Department of Epidemiology & Health Promotion occupies a 5,000 square foot area with 16 locked offices and 16 carrels. NYU Nursing facility has a 10,000 square foot state of the art simulation center for training the Registered Nurses. We will use this facility for clinical training, calibration, and standardization.

DISSEMINATION AND IMPLEMENTATION POTENTIAL

A. Potential for disseminating and implementing the results of this research in other settings *(PC-4.)*

Our proposal will support the NY City dissemination and implementation of study results through a concentrated, collaborative effort with our stakeholder partners, the NYC Department of Education, and the NYC Department of Health. We will report results from implementation experiences and outcome analyses in a digestible format to a diverse array of stakeholders and frame results in context of the larger clinical and policy implications for children and stakeholder organizations. We will pursue nation-wide dissemination through our relationships with Children's Health Fund and Univision.

The program proposed has the potential for a three-stage dissemination and implementation process: local, city, and national. The immediate, local, program-based D&I plan is to convey results on oral health surveillance and program impact to the 60 participating schools receiving simple or complex prevention. The mid-term plan is to expand to the larger NYC school system, as indicated in Section E (Engagement Plan) and the enclosed letter of support from Dr. De Stefano at the NYC Department of Education. In integrating our local and city-level D&I plan, we adopt the PCORI dissemination and implementation framework. For each outcome (caries arrest, prevention, academic performance, and quality of life) we will review the evidence of assessments with primary stakeholders to determine suitability and responsivity to stakeholder needs and concerns. We will then determine, for each outcome, who are the suitable partners and what is the larger audience in disseminating the results. We will then work internally to determine the best method to disseminate findings to the larger NYC DOE community, and work with interested schools to support larger adoption of the proposed program and facilitate its implementation.

More broadly, systematic reviews of dissemination and implementation [151,152] articulate a framework with 5 domains with 39 sub-domains to improve probability of success (Consolidated Framework for Implementation Research - CFIR). The CFIR framework complements the PCORI framework, and both are reflected in the Engagement Plan. These 5 CFIR domains include: 1) intervention characteristics; 2) outer setting; 3) inner setting; 4) characteristics of individuals; and 5) process. Below we briefly summarize how the proposed program addresses all 5 domains; we do not address the 39 sub-domains. We first address NYC, then NY State (NYS), and then the U.S.

<u>Intervention characteristics</u>: As indicated in the Patient Centeredness, the proposed school-based caries prevention program: 1) adheres to all six quality aims of the Institute of Medicine; 2) has demonstrated efficacy as shown by systematic reviews of randomized controlled trials (Table 1); and 3) complex prevention has demonstrated effectiveness based on pilot studies [153].

<u>Outer setting:</u> National, state, and city assessments of clinical need (and minimal resources for care) all provide support for school-based caries prevention. National and state policies also support school-based prevention. In NY State and NYC, this support is documented in the letters from the NYS DOH, the NYC DOE, and the NYC DOHMH.

Similarly, our national members of the External Advisory Board are interested in the program and its outcomes. These comments are verified in support letters from national and state dental leaders and dental insurers.

Inner setting: We are working within the NYC school system to identify "thought leader" principals who view oral health as an important component of child health and development, and want to participate. This is verified in support letters from the NYC DOE and the list of Bronx principals that want to participate.

Characteristics of individuals: There are two perspectives here: the parents and children. From both perspectives, the elementary school is an ideal setting for learning health behavior self-efficacy, along with all the other learning skill sets, over a multi-year time frame.

Process: In creating the proposed program, we conducted planning and engagement with parent and stakeholder partners. We are also incorporating community members in our research and oversight teams. This planning and engagement is indicated in support letters from the NYC DOE and DOHMH.

At the city level, we were asked to deliver care to all 1,600 schools in NYC. We agreed to start with 1,000 schools in order to collect gather evidence of effectiveness, and then develop plans to scale up to all schools. With the participation of the city and state DOE and DOH in this PCORI proposal, dissemination beyond the grant period will be limited only by the ability to solicit seed funding for expansion. The clinical portion of the proposed program is expected to be self-sufficient within the time context of the PCORI program. The design and implementation team, having developed and supported programs first in Massachusetts, and then in Colorado, Hawaii, Kansas, Maine, and New Hampshire, are adept at scaling infrastructure to deliver school-based carries prevention. The participation of Drs. Platt and De Stefano and Ms. Shah will facilitate dissemination in NYC. At the state level, participation in this application by Drs. Kumar and Gosling will facilitate dissemination across NY State.

To this end, we are working toward a national initiative to reach a "tipping point" with 20% of U.S. schoolchildren participating in comprehensive caries prevention programs. We have begun discussions with representatives of the ten largest U.S. school districts, which educate approximately 20% of U.S. schoolchildren. Our goal is to determine their interest in a national collaborative effort to provide a comprehensive school-based dental caries prevention program nation-wide. We also have spoken with school representatives from Puerto Rico, Hillsborough FL, and Hawaii, which are the 3rd, 9th, and 10th largest school districts after NYC), to gauge their interest in participating in such a program. All have spoken with their respective DOEs and all are interested in participating.

B. Describe possible barriers to disseminating and implementing the results of this research in other settings.

There are six potential barriers to implementation, none of which are technical. They are all financial, political or emotional: 1) financing for start-up; 2) identification of willing city and state partners in the DOE and DOH; 3) willing principals; 4) parents who sign informed consent; 5) state dental practice acts that allow dental hygienists to deliver care (still not the case in all states); and 6) Medicaid compensation to ensure sustainability. Previously, we addressed all of these issues in the states where we have worked and in NYC. Thus, we believe that these factors will be surmountable.

There are a number of problems in implementing evidence based medicine, as well as possible solutions. The size and complexity of research and difficulties in developing policy that reflects findings from evidence based medicine; poor access to best evidence; organizational behaviors, and low patient adherence to treatments can all restrict the efficient translation of study findings into practice. To facilitate dissemination of results, we adopt a clinical decision pathway: generate evidence from research, synthesize evidence, develop evidence based policy, and apply policies. In each step in this pathway, the intersection of patient circumstances, evidence of effectiveness, and patient wishes are leveraged. We will use known solutions to healthcare dissemination barriers, such as developing effective strategies in encouraging patients to follow advice and improve effectiveness of educational programs for interested stakeholders.

C. Describe how you will make study results available to study participants after you complete your analyses.

To lay the groundwork for implementation, we will disseminate the results of our work through local and national conferences and professional publications. For local and city level dissemination of results, we will convene yearly conferences with school principals to present findings on the state of oral health of their children and discuss results of program impact. We will also distribute bi-yearly newsletters to principals and parents regarding the program. In coordinating this outreach, we will designate a member of the team to be responsible for routine communication of the

program process and findings, and develop a strategy and timeline for press releases and articles. We will also make use of our departmental media coordinator to take advantage of social media and disseminate findings to school and DOE channels, such as PTA newsletters, school nurse communications, etc. Finally, we will use mass media opportunities through Univision TV/radio/print coverage, and the NYU College of Dentistry quarterly publication, the Nexus, which has a distribution of 40,000 among oral health policy makers, educators, and clinicians and the IESP newsletter goes out to >1,000 policy makers and educators. These media and publication sources will be ideal outlets for publicizing this program. Finally, we will take full advantage of the NYU press office to coordinate press releases about key findings.

To facilitate dissemination, we adopt a conceptual framework for improving dissemination of best practices. Under this framework, dissemination impact depends on contextual factors such as the innovation being tested and features of adopting organizations. Greater use of contextual considerations leads to more effective dissemination and greater adoption of evidence-based practice. These solutions include: providing simple, evidence-based recommendations; aligning messages with strategic goals of the participating organizations; engaging a sponsor for dissemination with credibility with participating organizations; providing practical implementation tools; creating networks to foster learning opportunities; and monitoring dissemination processes [154].

PROTECTION OF HUMAN SUBJECTS

In initial discussions with Institutional Review Boards (IRB) at NYU and the NYC DOE and DOHMH, there is informal agreement that the proposed school-based caries prevention program is exempt from Human Subjects Protection concerns because it provides the standard of care as described in 45CFR 46.101(b)(5). However, we will proceed with a formal NYU and NYC IRB approval process.

1 Risks to the subjects

1.1 Human Subjects Involvement and Characteristics

Program Characteristics. All students enrolled in the participating elementary schools, with informed consent, will receive caries prevention currently recommended by systematic reviews and national guidelines. Care will be provided independent of a family's ability to pay. As per NYC Department of Education consent guidelines, once a child consents to participation, that consent is valid as long as the child remains enrolled in the school.

Two types of care will be provided twice per year: simple prevention or complex prevention, as indicated in the Research Strategy. Simple prevention is defined as fluoride varnish and silver diamine fluoride. Complex prevention is defined as traditional sealants, therapeutic sealants, and fluoride varnish. Participants will be randomized at the school level to receive either simple or complex prevention. All participants will receive prevention twice a year. At the beginning of each school year, new students will be given consent forms and program information packets to take to parents, who will sign consent forms and return them along with other school permission or consent forms.

Ethnic and Racial Distribution. Schools were purposefully selected to have high Hispanic/Latino and low-SES populations. Schools were first sorted by race/ethnicity to identify schools with ≥ 50% Hispanic/Latino student population and then further restricted to schools with ≥ 80% of students receiving free or reduced lunch (the NYC DOE indicator for low-SES).

Inclusion and Exclusion Criteria. We will institute a two-level inclusion criterion: 1) students who attend the participating schools; and 2) students in these schools who provide informed consent and assent to participate in the study.

1.2 Sources of Materials

Sources of Research Material. The DOE will provide demographic and educational data on participating students in the participating schools. The clinical team will collect data as part of the routine dental examination, and as well preventive treatment by subject, tooth, or tooth surface at each visit. Clinical data will be derived from clinical examination by standardized examiners. Educational data will come from records maintained at the Office of School Health, housed at the NYC Department of Health and the NYC Department of Education. These records contain individual-level data for a complete census of K-12 students attending NYC public schools from the 1995-96 through 2016-17 academic years (a universe of roughly 1,000,000 observations per year) and for this project it will be current through 2022-2023. Every student record contains detailed demographic, program and academic information including birthplace, race, gender, language ability, attendance rates, participation in school breakfast and lunch, admit/discharge dates and codes, participation in special education and language programs, and standardized test scores in English Language Arts and Mathematics for students in grades 3-8. We will have data on students in both the experimental and the comparator group. These student-level data are combined with publicly available data from the *Annual School Reports, School Based Expenditure Reports* and *State Report Cards*, prepared annually by the NYC DOE and the New York State Education Department. There are no specialized resources or capabilities required to analyze the above data.

Biological Specimens. No biological specimens will be collected.

1.3 Potential Risks

The potential risks are minimal. These include breach of confidentiality and emotional challenge of care receipt. All preventive care is recommended by systematic reviews and national guidelines. Therefore, potential physical risks are no more or less than children receiving preventive care in a dental office. The risk of breach of confidentiality requires continuing attention throughout this project. The potential emotional risks, in contrast to physical and

confidentiality risks, are far less than those in a dental office because the children will be cared for in their own environment with friends nearby.

Any child identified in oral examinations as having serious dental maladies, such as sepsis or periodontal disease, will be referred to local dentists for follow-up care.

2 Adequacy of protection against risks

2.1 Recruitment and Consent

Recruitment will go in concert with all other school notifications and requests for treatment consent via informational packets sent home with the children to their parents/guardians. Dental packets will contain care details, informed consent form, HIPAA privacy form, and medical and oral health history form**.** Parents will be asked to return the forms to the child's school. These forms will be provided in multiple languages, depending on the school. The NYC DOE has a team of translation specialists for this purpose. We expect the average enrollment rates over the 5 years to be approximately 66%.

2.2 Protection Against Risk

Maintaining confidentiality is of the utmost importance and will be carefully protected. The study dental personnel will have access to the medical history and treatment documents. All subjects will be assigned a subject number. The Principal Investigators, Co-Investigator, and Program Manager will have access to identifying data. All of these individuals are HIPAA-trained and are bound by confidentiality agreements. All confidential forms and computers used for data entry and analysis will be stored in a locked file cabinet within locked rooms at NYU. No computers, datasheets or clinical forms will be overnight in any elementary school. All forms with personal or confidential information will be shredded after completion of the study analysis. Data will be input into the data system using subject numbers. Security at the Boston University Data Coordinating Center (DCC), including its computer systems, is carefully controlled by physical barriers and standard practices and procedures. DCC staff has user-defined roles and are password secured. Both the DCC and NYU have firewalls for their computer networks.

3 Potential benefits of the proposed research to the subjects and others

The benefits to participants in caries prevention program include: in-school dental examination, preventive care, and referral to a dentist. Successful completion of this project will provide important tactical and strategic benefits, locally and nationally. Locally, it will provide clinical assessments to support NYC dissemination. Nationally, it will provide an open-access database to test hypotheses about caries prevention strategies.

Ultimately, evidence-based changes in knowledge, attitudes and practice among all patient and stakeholder partners will help reduce the continually high burden of caries in children, particularly among minority and low-SES children. Improved outcomes data should increase support for the sustainability of ongoing and new caries prevention programs at both a local and national level. The benefits to society include improved oral health for our children that is sustained by a system of preventive care that is more clinically and cost effective than the current method of care.

4 Data and Safety Monitoring Plan

The proposed program will implement methods and materials that: 1) are currently used clinical practice; 2) were previously validated for safety and efficacy in randomized human controlled trials; and 3) are recommended by national guidelines, including the Centers for Disease Control and Prevention. The NYU Institutional Review Board has declared that a Data Safety Monitoring Board (DSMB) is not necessary. However, we will utilize a standard Data and Safety Monitoring Plan (DSMP).

Informed consent. Each participating school will obtain consent in accordance with their local circumstances. Because this project is designed to collect data to evaluate the currently delivered standard of care, the preliminary assessment by the NYU, NYC DOE, and DOHMH IRBs was that this study is exempt under 45 CRF46.101(b)(5). If funded, we will complete a formal IRB application.

Risk. Risks associated with program participation include potential loss of privacy of the data. The strict security and Data Monitoring plan outlined in this proposal will help reduce this potential risk. Monitoring will be carried out by the PIs and Program Director.

Implementation. On a daily basis, performance sites will upload data to the DCC. The DCC will maintain records for each performance site that includes patients seen and data. The DCC will provide weekly reports to the PIs, Program Director, and clinical research associate for each performance site that includes the daily reports. These reports will be matched with the MOPs to ensure timely recruitment and data reporting. If results are out of expected range, the clinical research associate will contact the clinical team to determine the cause and develop a plan to resolve the problem. Monthly, quarterly, and yearly reports will be generated by the DCC and provided to the PIs and Program Director. The initial reports will include recruitment data, while subsequent reports will include recruitment, retention, and databases for analysis.

5 Importance of the knowledge to be gained

As indicated in the Research Strategy, numerous efficacy trials demonstrate the benefits of complex preventive dental care, which has far-reaching effects on physical and emotional health, nutrition, social interactions and employability. However, numerous barriers still exist to improvements in child oral health: an already-existing global and national burden of caries; the burden of disease in NYC, particularly among Hispanic/Latino children from low-income families; the social burden of disease, particularly affecting academic performance and psycho-social development; barriers to office-based care; economic incentives to support treatment rather than prevention; the reduced efficacy of treatment to prevention; and large variation in care without outcome measures of effectiveness.

Without data to support the effectiveness of preventive oral health care, we predict that two things will occur (depending on state and local values and circumstances): 1) the current helter-skelter implementation of well-meaning but ineffective programs will continue; and/or 2) there will be an extinction of those public caries prevention programs that are truly effective.

From a national perspective, our preliminary data suggest that a school-based caries prevention program may represent the first program that exceeds the goals of *Healthy People 2000* [155]. If this happened, it would be transformative. This information would: 1) set the stage for broader dissemination within NYC and other metropolitan areas; and 2) stimulate implementation tests of other “place-based” caries prevention programs for other populations (e.g.: mothers, elderly, etc.), in locations where people learn, work, play, and pray.

Long term, we believe that children (and adults) with improved oral health will be better prepared to learn, thrive and improve their social and economic opportunities. These important accomplishments would be a first step in reducing social inequalities. The proposed school-based, cluster, randomized controlled trial, comparing two evidence-based caries prevention protocols, will address and potentially obviate all of the identified gaps.

Education of Key Personnel on the Protection of Human Subject Participants

All participating members of the research team will receive Protecting Human Research Participant training offered by the NIH Office of Extramural Research (OER). Any participating investigator who has received Collaborative Institute Training Initiative (CITI) approval will be considered to have satisfied the human subject research training requirement.

CONSORTIUM CONTRACTUAL ARRANGEMENTS

The single consortium arrangement is with the Boston University Data Coordinating Center. The Data Coordinating Center is the intermediary connecting the clinical care delivery team (represented by the Clinical Care Coordinator), and the multiple Analytics teams. In this context, the Data Coordinating Center is an essential participant in the current PCORI application. A letter of collaborative support is included in the Appendix.

More specifically and concretely, the clinical care delivery team uses electronic dental records and uploads them to the Data Coordinating Center at the end of each day. The Data Coordinating Center validates, cleans, and provides formatted data to the analytics teams.

The PI for this PCORI application and the Data Coordinating Center have been collaborating for more than 10 years on caries prevention programs. We began in Massachusetts in 2003, and are now collaborating on an NIH funded assessment and improvement initiative for rural school-based caries prevention programs. Together we evolved through multiple clinical recording, cleaning, and analysis protocols. We began with paper Teleform records and moved through electronic tablets to the current iPads. Throughout this evolution, we continuously linked records to facilitate patient centered data for longitudinal analysis. Samples of this are included in our preliminary results.

Collecting common data elements longitudinally across populations in multiple schools will allow us to make key comparisons around the primary outcome variable of oral health improvement (e.g.: caries arrest, untreated caries across program semester and within a particular child). And, it will allow us to compare academic performance and quality of life over time across schools.

To insure HIPAA compliance, identifiers will be removed and replaced with indicator variables. The Data Coordinating Center will create comprehensive data dictionaries, including documentation of all derived variables. Multiple datasets will be created in multiple formats (e.g.: SAS and Stata for analysis, and comma separated values (CSV files) for easy import into Excel) depending on the Analytic team's needs.

The Data Coordinating Center team includes Christine Chaisson, MPH, Principal Investigator of the Subcontract who will supervise all Data Coordinating Center personnel and activities to insure that all datasets are clean, fully de-identified, and accurate. Joseph Palmisano, MPH, MS, Statistical Analyst is a proficient SAS programmer and data analyst, and is the analyst for the project. He will create de-identified dataset which will entail removing all identifiers including dates which will be replaced by year/semester indicators and number of days between visits for instances when a child has more than one visit in a calendar year. Final de-identified datasets will be created in SAS and Stata and will be available as CSV files if requested. Leah Forman, MPH, Data Manager**,** is an experienced data manager and SAS programmer and serves as the data manager on the project. John Lu, MS, Systems Analyst**,** is Assistant Director for Web Applications at the DCC and is a proficient web and database programmer. Mr. Lu is the systems analyst on the project.

REFERENCES CITED

1. U.S. C. Bronx County, NY. U.S. Census Bureau. 2016. http://www.census.gov/search-results.html?q=bronx+ny&search.x=0&search.y=0&search=submit&page=1&stateGeo=none&searchtype=web
2. NYC. Data from: NYC School Metrics. 2016. *New York, NY*. Deposited 2016.
3. De Stefano M. NYC Department of Education, Personal Communication. 2014.
4. Casnoff CA, Noriega D, Estefan LF. Reserch Prioritization Topic Brief 5: Preventing Dental Caries in Children. PCORI. 2016. http://www.pcori.org/sites/default/files/PCORI-IHS-Advisory-Panel-Pre-Meeting-Topic-Briefs-011415.pdf
5. Cruz GD, Chen Y, Salazar CR, Karloopia R, LeGeros RZ. Determinants of oral health care utilization among diverse groups of immigrants in New York City. *J Am Dent Assoc*. Jul 2010;141(7):871-8.
6. Niederman R, Huang SS, Trescher A-L, Listl S. Universal Pre-K-8 School-Based Caries Prevention. *American Journal of Public Health*. 2017 (in press)
7. Dye B, Li X, Thornton-Evans G. Oral health disparities as determined by selected Healthy People 2020 Oral Health objectives for the United States, 2009-2010. *NCHS Data Brief*. 2012;104:1-8.
8. Dye BA, Thornton-Evans G, Li X, Iafolla TJ. Dental caries and sealant prevalence in children and adolescents in the United States, 2011-2012. *NCHS data brief*. 2015;(191):1-8.

9. Griffin SO, Wei L, Gooch BF, Weno K, Espinoza L. Vital Signs: Dental Sealant Use and Untreated Tooth Decay Among U.S. School-Aged Children. *MMWR Morbidity and Mortality Weekly Report ePub: 18 October 2016*. 2016;
10. Hosseinipour N, Jasek J, Summers C. Oral Health in New York City. *NYC Vital Signs* 2012;11(5):1-4.
11. Tellez M, Gray SL, Gray S, Lim S, Ismail AI. *Sealants and dental caries: dentists' perspectives on evidence-based recommendations*. Vol. 142. 2011:1033-40. *J Am Dent Assoc*. 1943-4723 (Electronic)
0002-8177 (Linking). Sep.
12. Annual EPSDT Participation Report Form CMS-416, Fiscal year: 2013 (U.S. Department of Health and Human Services, Centers for Medicaid and Medicare Services.) (2014).
13. Griffin SO, Barker LK, Wei L, et al. Use of dental care and effective preventive services in preventing tooth decay among U.S. Children and adolescents--Medical Expenditure Panel Survey, United States, 2003-2009 and National Health and Nutrition Examination Survey, United States, 2005-2010. *Morbidity and mortality weekly report Surveillance summaries*. Sep 12 2014;63 Suppl 2:54-60.
14. NNOHA. *Survey of School-Based Oral Health Programs Operated by Health Centers*. 2014. 2014. http://www.nnoha.org/nnoha-content/uploads/2014/07/SBHC-Report-FINAL_2014-07-28.pdf
15. CDHP. Dental Sealants: Proven to Prevent Tooth Decay. Children's Dental Health Project; 2014.
16. Niederman R, Guerrero D, Cugini M. Comparative Effectiveness of School-based Caries Prevention Programs: Baseline Assessment. *Journal of Dentistry*. 2017;(Submitted)
17. Marcenes W, Kassebaum NJ, Bernabé E, et al. Global burden of oral conditions in 1990-2010: a systematic analysis. *Journal of dental research*. 2013;92(7):592-7.
18. IOM. *Initial National Priorities for Comparative Effectiveness Research*. National Academies Press; 2009.
19. Pine CM, Harris RV, Burnside G, Merrett MC. An investigation of the relationship between untreated decayed teeth and dental sepsis in 5-year-old children. *British dental journal*. Jan 14 2006;200(1):45-7; discussion 29.
20. Otto M. For want of a dentist. *The Washington Post*. February 28, 2007. http://www.washingtonpost.com/wp-dyn/content/article/2007/02/27/AR2007022702116.html
21. Cruz GD, Chen Y, Salazar CR, Le Geros RZ. The association of immigration and acculturation attributes with oral health among immigrants in New York City. *Am J Public Health*. Oct 2009;99 Suppl 2:S474-80. doi:10.2105/ajph.2008.149799
22. Farber J. *Oral Health and the Commonwealth's Most Vulnerable Children: A State of Decay*. 2004.
23. Blumenshine SL, Vann WF, Jr., Gizlice Z, Lee JY. Children's school performance: impact of general and oral health. Research Support, Non-U.S. Gov't
Research Support, U.S. Gov't, Non-P.H.S.
Research Support, U.S. Gov't, P.H.S. *J Public Health Dent*. Spring 2008;68(2):82-7. doi:10.1111/j.1752-7325.2007.00062.x
24. Seirawan H, Faust S, Mulligan R. The impact of oral health on the academic performance of disadvantaged children. *Am J Public Health*. Sep 2012;102(9):1729-34. doi:10.2105/AJPH.2011.300478
25. Detty AM, Oza-Frank R. Oral health status and academic performance among Ohio third-graders, 2009-2010. *J Public Health Dent*. Jun 25 2014;doi:10.1111/jphd.12063
26. 31% of U.S. parents say kids missed school due to dental problems. Delta Dental Plans Association; July 15, 2015, 2015. http://www.prnewswire.com/news-releases/31-of-us-parents-say-kids-missed-school-due-to-dental-problems-300113340.html
27. Quinonez RB, Keels MA, Vann WF, Jr., McIver FT, Heller K, Whitt JK. Early childhood caries: analysis of psychosocial and biological factors in a high-risk population. *Caries Res*. Sep-Oct 2001;35(5):376-83. doi:47477
28. Mattheus DJ. Vulnerability related to oral health in early childhood: a concept analysis. *Journal of advanced nursing*. Sep 2010;66(9):2116-25. doi:10.1111/j.1365-2648.2010.05372.x

29. Finlayson TL, Siefert K, Ismail AI, Sohn W. Psychosocial factors and early childhood caries among low-income African-American children in Detroit. *Community dentistry and oral epidemiology*. Dec 2007;35(6):439-48. doi:10.1111/j.1600-0528.2006.00352.x
30. Adair PM, Burnside G, Pine CM. Analysis of health behaviour change interventions for preventing dental caries delivered in primary schools. *Caries Res*. 2013;47 Suppl 1:2-12. doi:10.1159/000351829
31. CMS. Keep Kids Smiling: Promoting Oral Health Through the Medicaid Benefit for Children & Adolescent. Center for Medicaid & Medicare Services. 2016. https://www.medicaid.gov/Medicaid/Benefits/Downloads/Keep-Kids-Smiling.pdf
32. Fisher-Owens SA, Gansky SA, Platt LJ, et al. Influences on children's oral health: a conceptual model. *Pediatrics*. Sep 2007;120(3):e510-20. doi:10.1542/peds.2006-3084
33. Sisson KL. Theoretical explanations for social inequalities in oral health. *Community dentistry and oral epidemiology*. Apr 2007;35(2):81-8. doi:10.1111/j.1600-0528.2007.00354.x
34. Nicolau B, Marcenes W, Bartley M, Sheiham A. A life course approach to assessing causes of dental caries experience: the relationship between biological, behavioural, socio-economic and psychological conditions and caries in adolescents. *Caries Res*. Sep-Oct 2003;37(5):319-26. doi:72162
35. Patrick DL, Lee RS, Nucci M, Grembowski D, Jolles CZ, Milgrom P. Reducing oral health disparities: a focus on social and cultural determinants. *BMC oral health*. 2006;6 Suppl 1:S4. doi:10.1186/1472-6831-6-s1-s4
36. Duijster D, van Loveren C, Dusseldorp E, Verrips GH. Modelling community, family, and individual determinants of childhood dental caries. *Eur J Oral Sci*. Apr 2014;122(2):125-33. doi:10.1111/eos.12118
37. ADA. Breaking Down Barriers to Oral Health for All Americans: The Role of the Workforce. Chicago IL: American Dental Association; 2011.
38. ADA. Action for Dental Health: Bringing disease prevention into communities. American Dental Association; 2013.
39. Isman B. *First 5: Oral Health Resource Guide*. First 5 California; 2014. Accessed July 2015. http://first5oralhealth.org/rural_smiles/downloads/chapter1/Potential%20Barriers.pdf
40. Vujicic M. Time to rethink dental "insurance". *Journal of the American Dental Association*. 2016;147(11):907-910.
41. Solomon ES. The past and future evolution of the dental workforce team. *J Dent Educ*. Aug 2012;76(8):1028-35.
42. CMS. National Health Expenditure Projections 2011-2021. U.S. Department of Health and Human Services. Accessed February 12, 2014, 2014.
43. HealthyPeople.gov. 2020 Topics & Objectives Oral Health. HealthyPeople.gov. Accessed Feb 12, 2014, 2014.
44. Webster AE, Johnson CN, Philadelphia PA. *Preparation of cavities for fillings*. 1909.
45. Downer MC, Azli NA, Bedi R, Moles DR, Setchell DJ. How long do routine dental restorations last? A systematic review. *British dental journal*. 1999;187(8):432-9.
46. Chadwick B, Treasure E, Dummer P, et al. Challenges with studies investigating longevity of dental restorations--a critique of a systematic review. *Journal of dentistry*. 2001;29(3):155-61.
47. Burke FJT, Lucarotti PSK. How long do direct restorations placed within the general dental services in England and Wales survive? *British dental journal*. 2009;206(1):E2; discussion 26-7.
48. HHS. *Promoting and Enhancing the Oral Health of the Public*. 2010.
49. CMS. Improving Access to and Utilization of Oral Health Services for Children in Medicaid and CHIP Programs. 2011;
50. Ricketts D, Lamont T, Innes NPT, Kidd E, Clarkson JE. Operative caries management in adults and children. *The Cochrane database of systematic reviews*. 2013;3:CD003808.
51. Schwendicke F, Dörfer CE, Paris S. Incomplete caries removal: a systematic review and meta-analysis. *Journal of dental research*. 2013;92(4):306-14.

52. Kohn LT, Corrigan J, Donaldson MS. *To err is human : building a safer health system*. National Academy Press; 2000:xxi, 287 p.
53. Niederman R. Bringing Care to People Rather Than People to Care. *Am J Public Health*. Jul 16 2015:e1. doi:10.2105/ajph.2015.302746
54. Niederman R, Feres M, Ogunbodede E. Dentistry. In: Debas HT, Donkor P, Gawande A, Jamison DT, Kruk ME, Mock CN, eds. *Disease Control Priorities Essential Surgery*. World Bank Group; 2015:173-195:chap 10.
55. Arrow P, Klobas E. Minimal Intervention Dentistry for Early Childhood Caries and Child Dental Anxiety: A Randomised Control Trial. *Australian dental journal*. Nov 23 2016;doi:10.1111/adj.12492
56. Arrow P, Klobas E. Child oral health-related quality of life and early childhood caries: a non-inferiority randomized control trial. *Australian dental journal*. Jun 2016;61(2):227-35. doi:10.1111/adj.12352
57. Niederman R. Bringing Care to People Rather Than People to Care. *Am J Public Health*. Sep 2015;105(9):1733. doi:10.2105/AJPH.2015.302746
58. Parnell C, Whelton H, O'Mullane D. Water fluoridation. *European archives of paediatric dentistry : official journal of the European Academy of Paediatric Dentistry*. 2009;10(3):141-8.
59. McDonagh MS, Whiting PF, Wilson PM, et al. Systematic review of water fluoridation. *BMJ (Clinical research ed)*. 2000;321(7265):855-9.
60. Griffin SO, Regnier E, Griffin PM, Huntley V. Effectiveness of fluoride in preventing caries in adults. *Journal of dental research*. 2007;86(5):410-5.
61. Johnson N, Lalloo R, Kroon J, Fernando S, Tut O. Effectiveness of water fluoridation in caries reduction in a remote Indigenous community in Far North Queensland. *Australian dental journal*. May 12 2014;doi:10.1111/adj.12190
62. Wong MCM, Clarkson J, Glenny AM, et al. Cochrane reviews on the benefits/risks of fluoride toothpastes. *Journal of dental research*. 2011;90(5):573-9.
63. Dos Santos AP, Nadanovsky P, de Oliveira BH. A systematic review and meta-analysis of the effects of fluoride toothpastes on the prevention of dental caries in the primary dentition of preschool children. *Community dentistry and oral epidemiology*. Aug 10 2012;doi:10.1111/j.1600-0528.2012.00708.x
64. Marinho VCC, Worthington HV, Walsh T, Clarkson JE. Fluoride varnishes for preventing dental caries in children and adolescents. *The Cochrane database of systematic reviews*. 2013;7:CD002279.
65. Mickenautsch S, Yengopal V. Caries-preventive effect of glass ionomer and resin-based fissure sealants on permanent teeth: An update of systematic review evidence. *BMC research notes*. 2011;4:22.
66. Yengopal V, Mickenautsch S, Bezerra AC, Leal SC. Caries-preventive effect of glass ionomer and resin-based fissure sealants on permanent teeth: a meta analysis. *Journal of oral science*. 2009;51(3):373-82.
67. Ahovuo-Saloranta A, Forss H, Walsh T, et al. Sealants for preventing dental decay in the permanent teeth. *The Cochrane database of systematic reviews*. 2013;3:CD001830.
68. de Amorim RG, Leal SC, Frencken JE. Survival of atraumatic restorative treatment (ART) sealants and restorations: a meta-analysis. *Clinical oral investigations*. 2012;16(2):429-41.
69. Rosenblatt A, Stamford TCM, Niederman R. Silver diamine fluoride: a caries "silver-fluoride bullet". *Journal of dental research*. 2009;88(2):116-25.
70. Zhi QH, Lo ECM, Lin HC. Randomized clinical trial on effectiveness of silver diamine fluoride and glass ionomer in arresting dentine caries in preschool children. *Journal of dentistry*. 2012;40(11):962-7.
71. Liu BY, Lo ECM, Chu CH, Lin HC. Randomized trial on fluorides and sealants for fissure caries prevention. *Journal of dental research*. 2012;91(8):753-8.
72. Niederman R, Gould E, Soncini J, Tavares M, Osborn V, Goodson JM. A model for extending the reach of the traditional dental practice: the ForsythKids program. *Journal of the American Dental Association (1939)*. 2008;139(8):1040-50.

73. Ruff RR, Palmisano J, Niederman R. Assessing Clinical Effectiveness of Rural Colorado School-Based Caries Prevention Programs. *BMC Oral Health*. 2017;(Submitted)
74. Mariño RJ, Khan AR, Morgan M. Systematic review of publications on economic evaluations of caries prevention programs. *Caries research*. 2013;47(4):265-72.
75. Källestål C, Norlund A, Söder B, et al. Economic evaluation of dental caries prevention: a systematic review. *Acta odontologica Scandinavica*. 2003;61(6):341-6.
76. Griffin SO, Naavaal S, Scherrer C, Patel M, Chattopadhyay S, Force CPST. Evaluation of School-Based Dental Sealant Programs: An Updated Community Guide Systematic Economic Review. *Am J Prev Med*. 2016;doi:10.1016/j.amepre.2016.10.004
77. Schwendicke F, Stolpe M, Meyer-Lueckel H, Paris S, Dörfer CE. Cost-effectiveness of one- and two-step incomplete and complete excavations. *Journal of dental research*. 2013;92(10):880-7.
78. Mickenautsch S, Yengopal V. Failure rate of high-viscosity GIC based ART compared with that of conventional amalgam restorations--evidence from an update of a systematic review. *SADJ : journal of the South African Dental Association = tydskrif van die Suid-Afrikaanse Tandheelkundige Vereniging*. 2012;67(7):329-31.
79. Mickenautsch S, Yengopal V, Banerjee A. Atraumatic restorative treatment versus amalgam restoration longevity: a systematic review. *Clinical oral investigations*. 2010;14(3):233-40.
80. Niederman R, Leitch J. "Know what" and "know how": knowledge creation in clinical practice. *Journal of dental research*. Apr 2006;85(4):296-7.
81. School-Based Oral Health Services: A Select Bibliography. Government/National Reports. Center for Health and Health Care in Schools. Accessed November 26, 2014, http://www.healthinschools.org/Health-in-Schools/Health-Services/School-Based-Dental-Health/Dental-Health-Bibliography.aspx
82. National Prevention Strategy. National Prevention Council, U.S. Department of Health and Human Services, Office of the Surgeon General. http://www.surgeongeneral.gov/initiatives/prevention/strategy/index.html
83. Oral Health Goals. U.S. Department of Health and Human Services, Office of the Surgeon General. 2015. http://www.healthypeople.gov/2020/topics-objectives/topic/oral-health
84. Educational & Community-Based Program Goals. U.S. Department of Health and Human Services, Office of the Surgeon General. http://www.healthypeople.gov/2020/topics-objectives/topic/educational-and-community-based-programs
85. IOM. *Children and Adolescent Health and Health Care Quality: Measuring What Matters*. The National Academies Press; 2011.
86. IOM. *Children and Adolescent Health and Health Care Quality: Measuring What Matters*. The National Academies Press; 2011.
87. Ahovuo-Saloranta A, Forss H, Hiiri A, Nordblad A, Mäkelä M. Pit and fissure sealants versus fluoride varnishes for preventing dental decay in the permanent teeth of children and adolescents. *Cochrane Database Syst Rev*. 2016;(1):CD003067. doi:10.1002/14651858.CD003067.pub4
88. Hilgert LA, Leal SC, Mulder J, Creugers NHJ, Frencken JE. Caries-preventive Effect of Supervised Toothbrushing and Sealants. *J Dent Res*. 2015;94(9):1218-24. doi:10.1177/0022034515592857
89. Bell RM, Klein SP, Bohannan HM, Disney JA, Graves RC, R. M. *Treatment Effects in the National Preventive Dentistry Demonstration Program*. 1984. http://www.rand.org/content/dam/rand/pubs/reports/2008/R3072.pdf
90. Klein SP, Bell RM. *Plan for the Analysis of Dental Examination Data in the National Preventive Dentistry Demonstration Program*. 1981. http://www.rand.org/content/dam/rand/pubs/notes/2009/N1658.pdf
91. Klein SP, Bohannan HM, Bell RM, Disney JA, Foch CB, Graves RC. The cost and effectiveness of school-based preventive dental care. *Am J Public Health*. Apr 1985;75(4):382-91.

92. Klein SP, Bohannan HM, Bell RM, Disney JA, Graves RC. Conjecture versus empirical data: a response to concerns raised about the National Preventive Dentistry Demonstration Program. *Am J Public Health*. Apr 1986;76(4):448-52.
93. Review of the National Preventive Dentistry Demonstration Program. *Am J Public Health*. Apr 1986;76(4):434-47.
94. Niederman R, Richards D, Brands W. The changing standard of care. *J Am Dent Assoc*. May 2012;143(5):434-7.
95. Niederman MS. *Severe pneumonia*. Lung biology in health and disease. Taylor & Francis; 2005:xv, 422 p.
96. Reference Manual on Scientific Evidence (3rd ed). In: Council FJCaNR, editor. Washington, DC: The National Academies Press; 2011.
97. Blumenthal-Barby JS, Krieger H. Cognitive biases and heuristics in medical decision making: a critical review using a systematic search strategy. *Med Decis Making*. 2015;35(4):539-57. doi:10.1177/0272989x14547740
98. de Vries EF, Struijs JN, Heijink R, Hendrikx RJP, Baan CA. Are low-value care measures up to the task? A systematic review of the literature. *BMC Health Serv Res*. 2016;16(1):405. doi:10.1186/s12913-016-1656-3
99. IOM. *Crossing the quality chasm : a new health system for the 21st century*. National Academy Press; 2001:337 p.
100. Frencken JE, Peters MC, Manton DJ, Leal SC, Gordan VV, Eden E. Minimal intervention dentistry for managing dental caries - a review: report of a FDI task group. *International dental journal*. 2012;62(5):223-43.
101. Joury E, Bernabe E, Sabbah W, Nakhleh K, Gurusamy K. Systematic review and meta-analysis of randomised controlled trials on the effectiveness of school-based dental screening versus no screening on improving oral health in children. *J Dent*. 2016;doi:10.1016/j.jdent.2016.11.008
102. AHRQ. National Guideline Clearing House. 2014. http://www.guideline.gov/index.aspx
103. De Stefano M. New York City, Department of Education. In: Niederman R, editor. 2014.
104. Improving Patient Safety Through Informed Consent for Patients with Limited Health Literacy. An implementation report. National Quality Forum; 2005. http://www.qualityforum.org/Publications/2005/09/Improving_Patient_Safety_Through_Informed_Consent_for_Patients_with_Limited_Health_Literacy.aspx
105. Implementing a National Voluntary Consensus Standard for Informed Consent. National Quality Forum; 2005. http://www.qualityforum.org/Publications/2005/09/Implementing_a_National_Voluntary_Consensus_Standard_for_Informed_Consent__A_User%E2%80%99s_Guide_for_Healthcare_Professionals.aspx
106. The AHRQ Informed Consent and Authorization Tooklit for Minimal Risk Research. (2009).
107. Broder HL, Wilson-Genderson M, Sischo L. Reliability and validity testing for the Child Oral Health Impact Profile-Reduced (COHIP-SF 19). *J Public Health Dent*. Fall 2012;72(4):302-12. doi:10.1111/j.1752-7325.2012.00338.x
108. Broder HL, Wilson-Genderson M, Sischo L. Oral health-related quality of life in youth receiving cleft-related surgery: self-report and proxy ratings. *Qual Life Res*. Oct 3 2016;doi:10.1007/s11136-016-1420-5
109. Ruff RR, Sischo L, Broder H. Resiliency and socioemotional functioning in youth receiving surgery for orofacial anomalies. *Community dentistry and oral epidemiology*. Aug 2016;44(4):371-80. doi:10.1111/cdoe.12222
110. Ruff RR, Sischo L, Broder HL. Validation of the Child Oral Health Impact Profile - Preschool version. *Community Dent Health*. 2017;In press

111. Loudon K, Treweek S, Sullivan F, Donnan P, Thorpe KE, Zwarenstein M. The PRECIS-2 tool: designing trials that are fit for purpose. *Bmj*. May 08 2015;350:h2147. doi:10.1136/bmj.h2147
112. Crystal YO, Niederman R. Silver Diamine Fluoride Treatment Consideration in Children's Caries Management. *Pediatric dentistry*. 2016;38 (in press)
113. Beltran ED, Malvitz DM, Eklund SA. Validity of two methods for assessing oral health status of populations. *J Public Health Dent*. Fall 1997;57(4):206-14.
114. White JA, Beltran ED, Malvitz DM, Perlman SP. Oral health status of special athletes in the San Francisco Bay Area. *Journal of the California Dental Association*. May 1998;26(5):347-54.
115. Petersen PE, Baez RJ. *Oral Health Surveys: Basic Methods*. World Health Organization; 2013.
116. Dye BA, Barker LK, Li X, Lewis BG, Beltran-Aguilar ED. Overview and quality assurance for the oral health component of the National Health and Nutrition Examination Survey (NHANES), 2005-08. *J Public Health Dent*. Winter 2011;71(1):54-61.
117. Lewis C, Lynch H, Richardson L. Fluoride varnish use in primary care: what do providers think? *Pediatrics*. 2005;115(1):e69-76. doi:10.1542/peds.2004-1330
118. Westat. *National Health and Nutrition Examination Survey III. Oral Examination Component*. Centers for Disease Control and Prevention; 1992.
119. NIH. *Oral health surveys of the National Insititue of Dental Research: Diagnostic Criteria and Procedures*. U.S. Department of Health and Human Services; 1991.
120. NIDCR. Toolkist for Clinical Researchers. Accessed July 7, 2014, 2014. http://www.nidcr.nih.gov/Research/toolkit/
121. Greene CJ, Morland LA, Durkalski VL, Frueh BC. Noninferiority and equivalence designs: issues and implications for mental health research. *J Trauma Stress*. Oct 2008;21(5):433-9. doi:10.1002/jts.20367
122. Sapra S. Semi-parametric mixed effects models for longitudinal data with applications in business and economics. *International Journal of Advanced Statistics and Probability*. 2014;2(2):84. doi:10.14419/ijasp.v2i2.3624
123. Sullivan KJ, Shadish WR, Steiner PM. An introduction to modeling longitudinal data with generalized additive models: applications to single-case designs. *Psychol Methods*. Mar 2015;20(1):26-42. doi:10.1037/met0000020
124. Shadish WR, Zuur AF, Sullivan KJ. Using generalized additive (mixed) models to analyze single case designs. *J Sch Psychol*. Apr 2014;52(2):149-78. doi:10.1016/j.jsp.2013.11.004
125. Vandormael A, Tanser F, Cuadros D, Dobra A. Estimating trends in the incidence rate with interval censored data and time-dependent covariates. *Stat Methods Med Res*. Jan 2020;29(1):272-281. doi:10.1177/0962280219829892
126. Hsu CH, Taylor JM, Murray S, Commenges D. Multiple imputation for interval censored data with auxiliary variables. *Stat Med*. Feb 20 2007;26(4):769-81. doi:10.1002/sim.2581
127. Pan W. A multiple imputation approach to Cox regression with interval-censored data. *Biometrics*. Mar 2000;56(1):199-203. doi:10.1111/j.0006-341x.2000.00199.x
128. Paul B, Sierra MA, Xu F, et al. Microbial population shift and metabolic characterization of silver diamine fluoride treatment failure on dental caries. *PLoS One*. 2021;16(3):e0242396. doi:10.1371/journal.pone.0242396
129. Ruff RR, Paul B, Sierra MA, et al. Predicting Treatment Nonresponse in Hispanic/Latino Children Receiving Silver Diamine Fluoride for Caries Arrest: A Pilot Study Using Machine Learning. *Front Oral Health*. 2021;2:695759. doi:10.3389/froh.2021.695759
130. Zhang J, Got S-R, Yin IX, Lo EC-M, Chu C-H. A Concise Review of Silver Diamine Fluoride on Oral Biofilm. *Applied Sciences*. 2021;11(7)doi:10.3390/app11073232
131. Abdullah N, Al Marzooq F, Mohamad S, et al. The antibacterial efficacy of silver diamine fluoride (SDF) is not modulated by potassium iodide (KI) supplements: A study on in-situ plaque biofilms using

viability real-time PCR with propidium monoazide. *PLoS One*. 2020;15(11):e0241519. doi:10.1371/journal.pone.0241519
132. Srisomboon S, Kettratad M, Pakawanit P, Rojviriya C, Phantumvanit P, Panpisut P. Effects of Different Application Times of Silver Diamine Fluoride on Mineral Precipitation in Demineralized Dentin. *Dent J (Basel)*. Jun 14 2021;9(6)doi:10.3390/dj9060070
133. Zhao IS, Gao SS, Hiraishi N, et al. Mechanisms of silver diamine fluoride on arresting caries: a literature review. *International dental journal*. Apr 2018;68(2):67-76. doi:10.1111/idj.12320
134. Peng JY, Botelho MG, Matinlinna JP, Pan HB, Kukk E, Low KJ. Interaction of storage medium and silver diamine fluoride on demineralized dentin. *J Int Med Res*. Feb 2021;49(2):300060520985336. doi:10.1177/0300060520985336
135. Firouzmandi M, Vasei F, Giti R, Sadeghi H. Effect of silver diamine fluoride and proanthocyanidin on resistance of carious dentin to acid challenges. *PLoS One*. 2020;15(9):e0238590. doi:10.1371/journal.pone.0238590
136. Jg B, S S, J D, N G. Predictor model of root caries in older adults: reporting of evidence to the translational evidence mechanism. *Open Dent J*. 2010;4:124-32. doi:10.2174/1874210601004010124
137. Khouja T, Smith KJ. Cost-effectiveness analysis of two caries prevention methods in the first permanent molar in children. *J Public Health Dent*. Mar 2018;78(2):118-126. doi:10.1111/jphd.12246
138. Kopycka-Kedzierawski DT, Billings RJ. Application of nonhomogenous Markov models for analyzing longitudinal caries risk. *Community dentistry and oral epidemiology*. Apr 2006;34(2):123-9. doi:10.1111/j.1600-0528.2006.00268.x
139. Burchett WW, Ellis AR, Harrar SW, Bathke AC. Nonparametric Inference for Multivariate Data: The R Package npmv. *Journal of Statistical Software*. 2017;76(4)doi:10.18637/jss.v076.i04
140. Wobbrock JO, Findlater L, Gergle D, Higgins JJ. The Aligned Rank Transform for Nonparametric Factorial Analyses Using Only ANOVA Procedures. 2011:
141. Benjamini Y, Hochberg Y. Controlling the False Discovery Rate: A Practical and Powerful Approach to Multiple Testing. *Journal of the Royal Statistical Society*. 1995;57(1):289-300.
142. Chow S, Shao J, Wang H. *Sample size calculations in clinical research* CRC Biostatistics Series. Taylor & Francis; 2008.
143. Diggle PJ, Heagerty P, Kung-Yee L, Zeger SL. *Analysis of Longitudinal Data*. Oxford Science Series. Oxford University Press; 2002.
144. Altman D, Hoey J, Marusic A, Moher D, Schulz KF. EQUATOR Network. http://www.equator-network.org/
145. Chan AW, Tetzlaff JM, Altman DG, et al. SPIRIT 2013 statement: defining standard protocol items for clinical trials. *Ann Intern Med*. Feb 5 2013;158(3):200-7. doi:10.7326/0003-4819-158-3-201302050-00583
146. Schulz KF, Altman DG, Moher D, Group C. CONSORT 2010 statement: updated guidelines for reporting parallel group randomised trials. *Bmj*. 2010;340:c332. doi:10.1136/bmj.c332
147. Campbell MK, Piaggio G, Elbourne DR, Altman DG, Group C. Consort 2010 statement: extension to cluster randomised trials. *Bmj*. 2012;345:e5661. doi:10.1136/bmj.e5661
148. Lang L, Altman D. Basic statistical reporting for articles published in clinical medical journals: the SAMPL Guidelines. In: Smart P, Maisonneuve H, Polderman A, eds. *Science Editors' Handbook*. European Association of Science Editors; 2013.
149. Hoffmann TC, Glasziou PP, Boutron I, et al. Better reporting of interventions: template for intervention description and replication (TIDieR) checklist and guide. *Bmj*. 2014;348:g1687. doi:10.1136/bmj.g1687
150. NYU Colldge of Dentistry to Partner with Univision to Promote Improved Oral Health Among Hispanics. 2016. http://www.nyu.edu/about/news-publications/news/2016/august/nyu-college-of-dentistry-to-partner-with-univision-to-promote-im.html

151. Damschroder LJ, Aron DC, Keith RE, Kirsh SR, Alexander JA, Lowery JC. Fostering implementation of health services research findings into practice: a consolidated framework for advancing implementation science. *Implementation science : IS*. 2009;4:50. doi:10.1186/1748-5908-4-50
152. Greenhalgh T, Robert G, Macfarlane F, Bate P, Kyriakidou O. Diffusion of innovations in service organizations: systematic review and recommendations. *The Milbank quarterly*. 2004;82(4):581-629. doi:10.1111/j.0887-378X.2004.00325.x
153. Niederman R, Gould E, Soncini J, Tavares M, Osborn V, Goodson JM. A model for extending the reach of the traditional dental practice: the ForsythKids program. Comparative Study

Multicenter Study

Research Support, Non-U.S. Gov't. *J Am Dent Assoc*. Aug 2008;139(8):1040-50.
154. Wilson PM, Petticrew M, Calnan MW, Nazareth I. Disseminating research findings: what should researchers do? A systematic scoping review of conceptual frameworks. *Implementation science : IS*. Nov 22 2010;5:91. doi:10.1186/1748-5908-5-91
155. HHS. Healthy People 2020. HHS. Accessed July 18, 2014, 2014. http://www.healthypeople.gov/2020/topicsobjectives2020/objectiveslist.aspx?topicId=32
