# Supplementary material for: Noninferiority of Silver Diamine Fluoride vs Sealants for Reducing Dental Caries Prevalence and Incidence: A Randomized Clinical Trial
Source: JAMA Pediatr. 2024 Mar 4;178(4):354–61. doi: 10.1001/jamapediatrics.2023.6770 (PMC10913007; doi:10.1001/jamapediatrics.2023.6770)
Supplement: Supplement 2. — eTable 1. Longitudinal caries incidence and the effect of sealants with ART compared to silver diamine fluoride, full sample eFigure. CONSORT diagram, COVID-19 sample eTable 2. Sample demographics overall and by treatment, COVID-19 sample eTable 3. Longitudinal caries prevalence and the effect of sealants with ART compared to silver diamine fluoride, COVID-19 sample eTable 4. Longitudinal caries incidence and the effect of sealants with ART compared to silver diamine fluoride, COVID-19 sample [file jamapediatr-e236770-s002.pdf]

Supplemental Online Content

Ruff RR, Barry Godín T, Niederman R. Noninferiority of silver diamine fluoride vs sealants for reducing dental caries prevalence and incidence: a randomized clinical trial. *JAMA Pediatr*. Published online February 26, 2024. doi:10.1001/jamapediatrics.2023.6770

eTable 1. Longitudinal caries incidence and the effect of sealants with ART compared to silver diamine fluoride, full sample

eFigure. CONSORT diagram, COVID-19 sample

eTable 2. Sample demographics overall and by treatment, COVID-19 sample

eTable 3. Longitudinal caries prevalence and the effect of sealants with ART compared to silver diamine fluoride, COVID-19 sample

eTable 4. Longitudinal caries incidence and the effect of sealants with ART compared to silver diamine fluoride, COVID-19 sample

This supplemental material has been provided by the authors to give readers additional information about their work.

© 2024 Ruff RR et al. *JAMA Pediatrics.*

eTable 1. Longitudinal caries incidence and the effect of sealants with ART compared to silver diamine fluoride, full sample

	IRR	SE	p-value	95% L	95% U
Observational period	0.828	0.009	< .001	0.810	0.846
Active control (vs exp)	0.924	0.053	0.172	0.825	1.035
Baseline decay	17.277	1.018	< .001	15.393	19.391
Previous care	0.758	0.076	0.005	0.624	0.922
Sex (male)	1.050	0.055	0.349	0.948	1.164
Race/Ethnicity					
Black	0.987	0.068	0.854	0.863	1.130
White	0.721	0.133	0.076	0.503	1.035
Asian	0.907	0.170	0.602	0.627	1.310
More than one	0.932	0.208	0.752	0.602	1.442
Other	1.742	0.304	0.001	1.237	2.452
Unreported	0.931	0.071	0.351	0.801	1.082

Notes: total number of teeth at each observation with untreated dental caries was the outcome. Includes all retained children enrolled in the CariedAway study.

© 2024 Ruff RR et al. *JAMA Pediatrics.*

eFigure. CONSORT diagram, COVID-19 sample

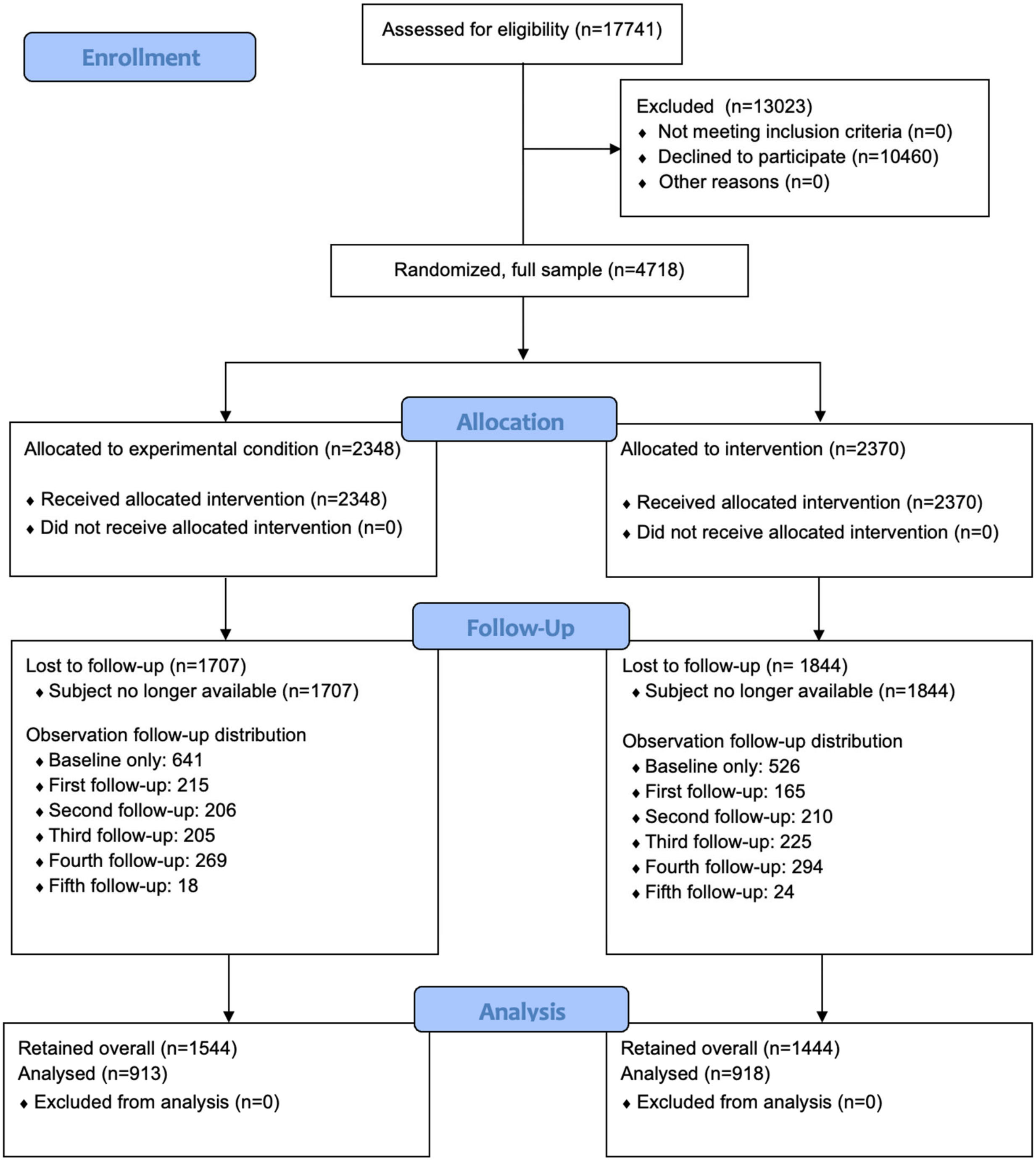

Notes: This CONSORT diagram reflects those subjects in the CariedAway trial who were enrolled, randomized, and treated in the six months prior to school shutdowns due to COVID-19. This subsample ensures that all children analyzed had the same elapsed time between observations.

© 2024 Ruff RR et al. *JAMA Pediatrics.*

eTable 2. Sample demographics overall and by treatment, COVID-19 sample

	Overall		Experimental		Active Control	
	N	%	N	%	N	%
Participants	2998	100	1554	51.83	1444	48.17
Baseline decay	874	29.15	482	31.02	392	27.15
Sex (male)	1432	47.77	664	45.98	769	49.42
Race/Ethnicity						
Hispanic	1631	54.4	810	52.12	821	56.86
Black	558	18.61	311	20.01	247	17.11
White	75	2.5	36	2.32	39	2.7
Asian	44	1.47	29	1.87	15	1.04
More than one	58	1.93	34	2.19	24	1.66
Other	41	1.37	24	1.54	17	1.18
Unreported	591	19.71	310	19.95	281	19.46
Age at baseline	6.63	1.24 (SD)	6.62	1.25 (SD)	6.65	1.24 (SD)

Notes: sample includes any subject that (1) was enrolled in the six-month data collection period prior to school shutdowns due to COVID-19 and (2) completed a follow-up observation upon resumption of study activities.

© 2024 Ruff RR et al. *JAMA Pediatrics.*

eTable 3. Longitudinal caries prevalence and the effect of sealants with ART compared to silver diamine fluoride, COVID-19 sample

	OR	SE	p	95% L	95% U
Observational period	0.789	0.025	< .001	0.741	0.84
Active control (vs exp)	1.04	0.125	0.763	0.819	1.31
Baseline decay	46.57	7.18	< .001	34.43	62.99
Previous care	0.744	0.152	0.148	0.499	1.11
Sex (Males)	1.04	0.125	0.751	0.821	1.31
Race/Ethnicity					
Black	1.04	0.158	0.797	0.772	1.4
White	0.89	0.342	0.757	0.418	1.89
Asian	0.489	0.224	0.118	0.2	1.2
More than one	0.663	0.363	0.454	0.227	1.94
Other	2.05	0.844	0.08	0.917	4.59
Unreported	1.07	0.205	0.723	0.735	1.56

© 2024 Ruff RR et al. *JAMA Pediatrics.*

eTable 4. Longitudinal caries incidence and the effect of sealants with ART compared to silver diamine fluoride, COVID-19 sample

	IRR	SE	p-value	95% L	95% U
Observational period	0.816	0.013	< .001	0.792	0.841
Active control (vs exp)	0.995	0.079	0.948	0.851	1.163
Baseline decay	12.678	0.106	< .001	10.770	14.930
Previous care	0.776	0.103	0.056	0.598	1.001
Sex (male)	1.030	0.078	0.651	0.893	1.120
Race/Ethnicity					
Black	1.000	0.097	0.964	0.832	1.213
White	0.878	0.217	0.598	0.540	1.426
Asian	0.629	0.187	0.119	0.351	1.127
More than one	0.728	0.244	0.345	0.377	1.406
Other	1.560	0.392	0.078	0.952	2.553
Unreported	0.981	0.117	0.873	0.776	1.240

© 2024 Ruff RR et al. *JAMA Pediatrics.*
