# Supplementary material for: Noninferiority of Silver Diamine Fluoride vs Sealants for Reducing Dental Caries Prevalence and Incidence: A Randomized Clinical Trial
Source: JAMA Pediatr. 2024 Mar 4;178(4):354–61. doi: 10.1001/jamapediatrics.2023.6770 (PMC10913007; doi:10.1001/jamapediatrics.2023.6770)
Supplement: Supplement 3. — Data sharing statement [file jamapediatr-e236770-s003.pdf]

Data Sharing Statement

Ruff. Noninferiority of Silver Diamine Fluoride vs Sealants for Reducing Dental Caries Prevalence and Incidence. *JAMA Pediatr*. Published February 26, 2024. doi:10.1001/jamapediatrics.2023.6770

Data
Data available: Yes
Data types: Data dictionary, Other (please specify)
Additional Information: De-identified data upon request and completion of a data use agreement
How to access data: Interested researchers should send data requests to the corresponding author, ryan.ruff@nyu.edu
When available: With publication

Supporting Documents
Document types: Informed consent form
How to access documents: Interested researchers should send data requests to the corresponding author, ryan.ruff@nyu.edu
When available: With publication

Additional Information
Who can access the data: Researchers whose proposed use of the data has been approved.
Types of analyses: For any purpose.
Mechanisms of data availability: After approval of a proposal and a signed data use access agreement.
